# Insights into Siglec‐7 Binding to Gangliosides: NMR Protein Assignment and the Impact of Ligand Flexibility

**DOI:** 10.1002/advs.202415782

**Published:** 2025-04-26

**Authors:** Cristina Di Carluccio, Luis Padilla‐Cortés, Marta Tiemblo‐Martìn, Giulia Roxana Gheorghita, Rosario Oliva, Linda Cerofolini, Alessandro Antonio Masi, Celeste Abreu, Hsin‐Kai Tseng, Antonio Molinaro, Pompea Del Vecchio, Ondřej Vaněk, Chun‐Cheng Lin, Roberta Marchetti, Marco Fragai, Alba Silipo

**Affiliations:** ^1^ Department of Chemical Sciences University of Naples Federico II Via Cinthia 4 Naples 80126 Italy; ^2^ CEINGE‐Biotecnologie Avanzate Franco Salvatore Via Gaetano Salvatore 486 Napoli 80145 Italy; ^3^ Magnetic Resonance Centre (CERM) CIRMMP and Department of Chemistry “Ugo Schiff” University of Florence Via Luigi Sacconi 6 Sesto Fiorentino 50019 Italy; ^4^ Giotto Biotech s.r.l. Sesto Fiorentino 50019 Italy; ^5^ Department of Biochemistry Faculty of Science Charles University Hlavova 2030/8 Prague 12800 Czech Republic; ^6^ Department of Chemistry National Tsing Hua University Hsinchu 300044 Taiwan; ^7^ Department of Medicinal and Applied Chemistry Kaohsiung Medical University Kaohsiung 80708 Taiwan

**Keywords:** gangliosides, NMR, siglec‐7, structural biology

## Abstract

Gangliosides, sialylated glycosphingolipids abundant in the nervous system, play crucial roles in neurotransmission, interaction with regulatory proteins, cell–cell recognition, and signaling. Altered gangliosides expression has been correlated with pathological processes, including cancer, inflammatory disorders, and autoimmune diseases. Gangliosides are important endogenous ligands of Siglecs (Sialic acid‐binding immunoglobulin‐type lectins), I‐type lectins mostly expressed by immune cells, that specifically recognize sialylated glycans. Siglec‐7, an inhibitory immune receptor on human natural killer cells, represents a potential target for tumor immunotherapy. Notably, the expression of Siglec‐7 ligands is high in various cancers, such as pancreatic cancer and melanoma and lead to tumor immune evasion. Siglec‐7 binds the disialylated ganglioside GD3, a tumor‐associated antigen overexpressed on cancer cells to suppress immune responses. Using a combination of structural biology techniques, including Nuclear Magnetic Resonance (NMR), biophysical, and computational methods, the binding of Siglec‐7 to GD3 and Gb3 derivatives is investigated, revealing the importance of ligand conformation in modulating binding energetics and affinity. The greater flexibility of Gb3 derivatives appears to negatively impact binding entropy, leading to lower affinity compared to GD3. A thorough understanding of these interactions could contribute to elucidating molecular mechanisms of cancer immune evasion and facilitate the development of ganglioside‐based diagnostic and therapeutic strategies for cancer.

## Graphical abstract

The molecular recognition of the sugar moiety of GD3 and Gb3 (globotriaosylceramide) derivatives by Siglec‐7 has been thoroughly studied offering insights for cancer diagnostics and therapies. A combination of structural biology techniques, nuclear magnetic resonance (NMR) spectroscopy, physicochemical analysis, and in silico methods suggested that Siglec‐7 recognition of gangliosides depends on their length and conformational flexibility.

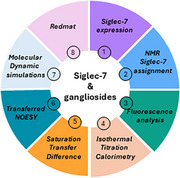

## 1 Introduction

Sialic acids (Sias) are a family of negatively charged nonulosonic acids attached to the terminal portion of *N*‐glycans, *O*‐glycans, and glycosphingolipids via α2‐3, α2‐6 and/or α2‐8 glycosidic linkages, resulting in an immense diversity.^[^ ^1^ ^]^ Moreover, sialoglycans recognition can be translated into a specific biological response when they are targeted by host receptors.^[^ ^2^ ^]^ Among these, Siglecs, sialic acid‐binding immunoglobulin‐like receptors, are I‐type lectins that share an *N*‐terminal Ig domain that recognizes sialic acid–containing glycans.^[^ ^3^ ^]^ They are expressed mainly by immune cells, including B‐cells, NK cells, eosinophils, neutrophils, and dendritic cells and play a crucial role in distinguishing between self and non‐self via glycan recognition.

Gangliosides are glycosphingolipids containing one or more sialic acids, whose lipidic part is composed of ceramide. Ceramides are made up of sphingosine and a fatty acid connected by an amide bond. The ganglio series (GgCer) tetrasaccharide core is formed by Gal*p*β1–3Gal*p*NAcβ1–4Gal*p*β1–Cer, with the length varying depending on the specific ganglioside. In addition to variations in the elongation of the core structure, gangliosides are characterized also by the number and positions of sialic acids which categorize them into 0, M, D, T, Q, and P (zero to five sialic acids) and a, b, and c (one, two, or three sialic acids on internal Gal residue) series. Specifically, in GD3, the “ganglio” core consists of four saccharide residues, denoted by the letter G, with the letter D indicating the presence of two sialic acid residues.^[^ ^4^ ^]^ They are endogenous Siglec ligands,^[^ ^5^ ^]^ structurally composed of an extra‐cellular carbohydrate moiety linked to ceramide, a hydrophobic lipid portion embedded in the membrane. Recently, the study of gangliosides has made significant advances, particularly concerning their role in pathological events. Even though they are broadly distributed, gangliosides predominate in the brain,^[^ ^6^ ^]^ and in particular, disialyl gangliosides crucially intervene in cellular processes such as neurotransmission, interaction with regulatory proteins, cell–cell recognition, and modulation of signal transduction pathways.^[^ ^7^, ^8^ ^]^ In the nervous system, GD1a and GT1b are recognized by Siglec‐4 (or myelin‐associated glycoprotein MAG) to support axon‐myelin interactions essential for long‐term axonal survival. Changes in gangliosides distribution and content are associated with malignancies; certain cancers also produce and shed gangliosides that have immunosuppressive effects. Disialylated gangliosides in cancers affect cell behavior, such as proliferation, migration, invasion, adhesion, and angiogenesis, as well as tumor immunosuppression.^[^ ^9^ ^]^ Siglec‐7 is an inhibitory immune receptor expressed on human natural killer (NK) cells and subsets of myeloid and dendritic cells, and represents a potential new target for tumor immunotherapy.^[^ ^10^, ^11^ ^]^ Sialoglycans expressed on cancer cells engage Siglec‐7 and inhibit NK cell‐mediated killing, thus making Siglec‐7 and cognate ligands novel glyco‐immune checkpoints for cancer immunotherapy.^[^ ^12^, ^13^ ^]^ Siglec‐7 preferentially binds internally branched α2,6‐linked disialylated gangliosides, such as DSGb5, disialosyl Lc4 (DSLc4), and α2,8‐linked gangliosides such as GD2, GD3, and GT1b.^[^ ^14^, ^15^ ^]^ GD3 (NeuAcα2‐8NeuAcα2‐3Galβ1‐4Glcβ1‐1′Cer) is crucial for brain development but its levels are decreased in adults.^[^ ^16^ ^]^ Conversely, GD3 is considered a crucial tumor‐associated antigen, being upregulated in pathological conditions, such as cancers and neurodegenerative disorders.^[^ ^17^, ^18^ ^]^ Once sialoglycans on cancer cells’ surface target Siglec‐7, the cancer cells evade immune detection and proliferate.^[^ ^19^, ^20^ ^]^ Recent findings indicate that cell surface GD3 with classical ceramide supports Siglec‐7 binding, whereas GD3 with ceramide alterations, including an extra hydroxyl group on sphingosine C4 (phytoceramide) or a 2‐hydroxyl group on the fatty acid amide, does not effectively engage with Siglec‐7. Understanding the dynamics of these interactions holds great promise for providing insights into disease mechanisms and potentially opening the door to developing diagnostic and therapeutic strategies.^[^ ^21^ ^]^ This can have significant implications in immunotherapy, where targeting these interactions may lead to novel therapeutic strategies against cancer, which often exploits sialoglycans to immune evasion mechanisms. To deeply understand Siglec‐7 binding to closely related sialoglycan structures, and with the long‐term goal of developing potential ligands within cancer immunotherapy, we undertook a comprehensive study of Siglec‐7 recognition and binding to the sugar portions of GD3^[^ ^22^ ^]^ and two Gb3 (globotriaosylceramide) derivatives,^[^ ^23^ ^]^ such as DSGb3α3 and DSGb3α6 (Scheme S1, Supporting Information). For this purpose, we employed a combination of multidisciplinary and complementary methods, consisting of fluorescence, high‐resolution ligand‐ and protein‐based Nuclear Magnetic Resonance (NMR) experiments, isothermal titration calorimetry (ITC), and computational approaches, including docking, molecular dynamics (MD) Ψ simulations, and RedMat 3D structure evaluation program^[^ ^24^ ^]^ to obtain information about binding preferences, affinities and 3D molecular features of such protein‐ligand complexes. Comprehending these interactions is instrumental in elucidating their roles in immunology, disease pathology, and potential therapeutic applications.

## 2 Results and Discussion

The molecular binding between the glycosylated Siglec‐7 and gangliosides (ligands GD3, DSGb3α3, and DSGb3α6, Figure S1 and Scheme S1, Supporting Information) was investigated using a combination of biophysical and computational methods.

### 2.1 NMR Assignment of the Backbone ^1^H and ^15^N Resonances of Siglec‐7 CRD

**Figure 1:**
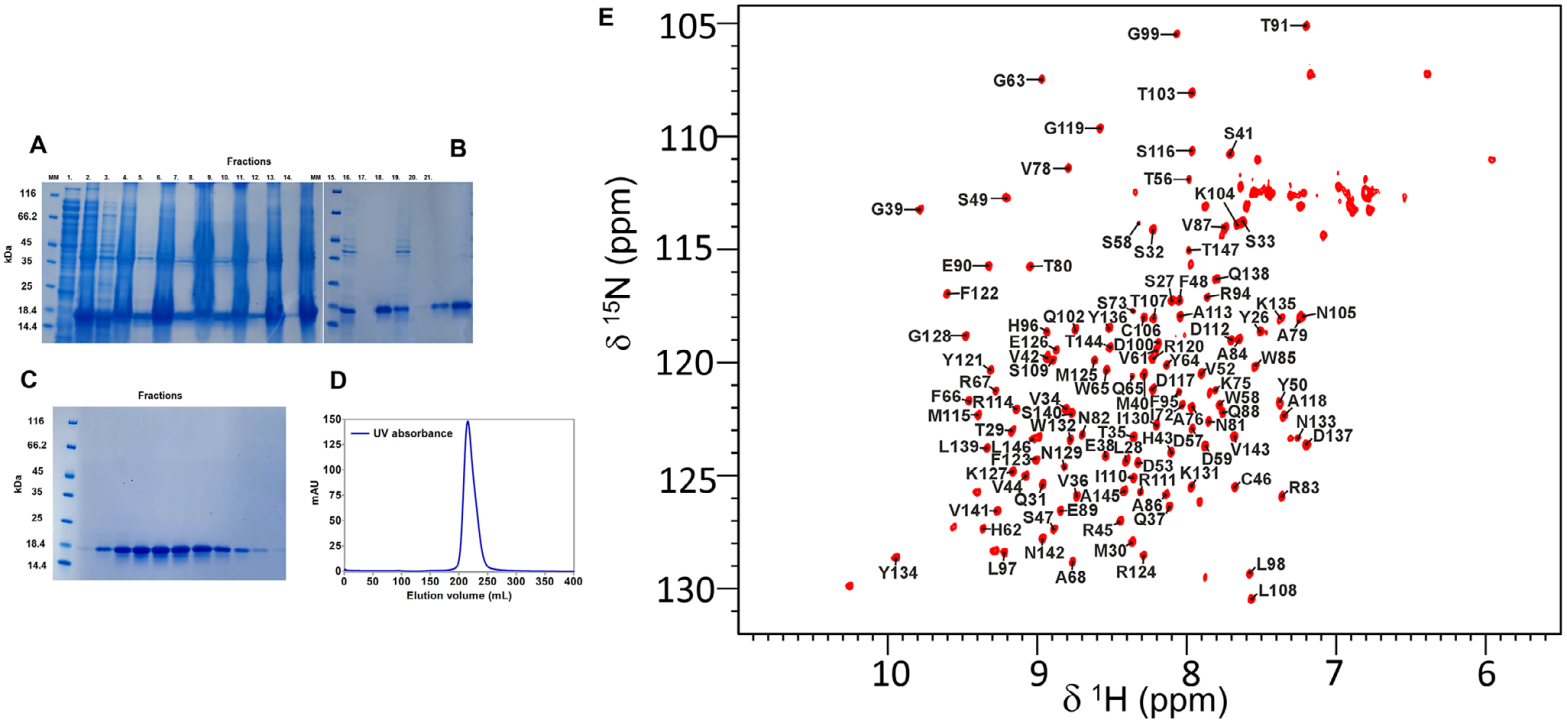
Siglec‐7 expression, purification, and backbone assignment by NMR. A) SDS‐PAGE representation: Lysis step – (1) supernatant, (2) cell debris; Solubilization of Inclusion bodies I‐ (3) supernatant, (4) cell debris; Solubilization of Inclusion bodies II – (5) supernatant, (6) cell debris; Solubilization of Inclusion bodies III – (7) supernatant, (8) cell debris; Solubilization of Inclusion bodies IV – (9) supernatant, (10) cell debris; Solubilization of Inclusion bodies V – (11) supernatant, (12) cell debris; Solubilization of Inclusion bodies VI – (13) supernatant, (14) cell debris. B) SDS‐PAGE representation: HisTrap purification, 1st cycle – Flow‐through (15), Column wash (16), Elution (17); HisTrap purification, 2nd cycle – Flow‐through (18), Column wash (19), Elution (20), 1st Elution and 2nd Elution (21). C) SDS‐PAGE representation: Size‐exclusion chromatography – collected fraction of Siglec‐7 CRD. D) Size‐exclusion chromatogram representation of Siglec‐7 CRD as single peak. E) 2D ^1^H‐^15^N HSQC NMR spectrum of the apo Siglec‐7 CRD in 20 mm KPi, 50 mm NaCl, pH 7.4 acquired on a spectrometer operating at 900 MHz at 298 K. NH amino acid resonances obtained by the protein assignment are reported in the spectrum.

The *N*‐terminal Ig‐like C‐type carbohydrate recognition domain (CRD) of Siglec‐7 (Scheme S1, Supporting Information) has been expressed in *E. coli* in M9 culture medium containing ^15^N NH_4_Cl and ^13^C‐glucose. The protein was recovered from inclusion bodies and after a refolding protocol, a highly pure isotopically enriched Siglec‐7 CRD was obtained. ^1^H, ^15^N, and ^13^C backbone resonances were assigned through solution‐state NMR. 2D ^1^H‐^15^N HSQC NMR experiments showed well‐dispersed resonances, which were used as confirmation of the folding state of the protein (**Figure** **1**). 3D triple‐resonance experiments HNCA, HNCACB, HNCO, and CBCAcoNH were acquired at 900 MHz, while HNcaCO on a spectrometer operating at 500 MHz. 93% of the amino acid sequence from Y26 to T147 was assigned, excluding the 5 proline residues and the histidine‐tag tail. The global folding of Siglec‐7 CRD was not changed by the presence of glycosylated sites.^[^ ^25^ ^]^ The isotopically enriched Siglec‐7 CRD from *E. coli* was then used in protein‐based NMR experiments for binding studies with gangliosides **1–3** (see below).

### 2.2 Ligand‐Binding Thermodynamics

**Figure 2:**
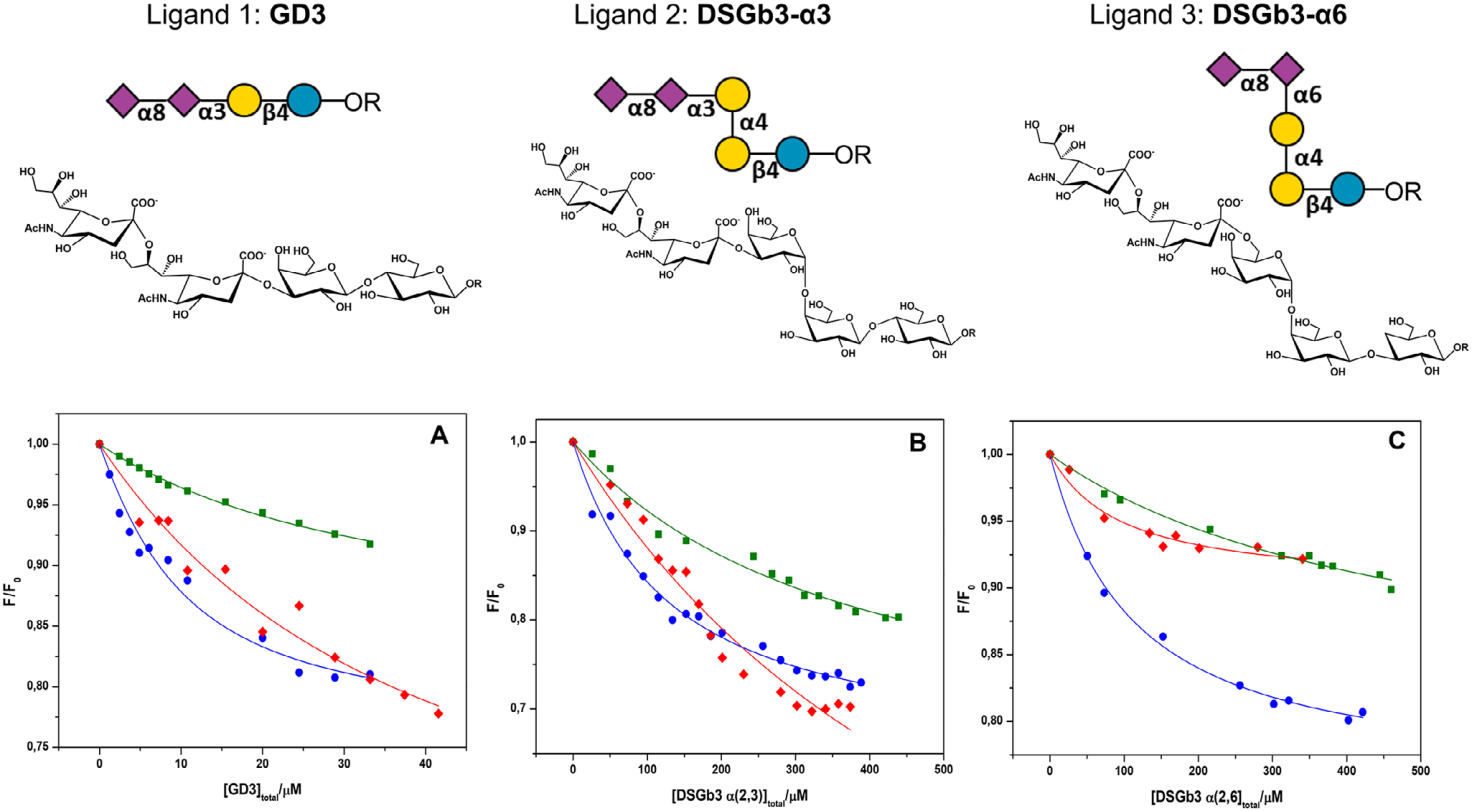
Binding isotherms obtained by means of fluorescence spectroscopy for the complex formation between Siglec‐7 CRD and (A) ligand **1** (GD3), (B) ligand **2** (DSGb3α3), and (C) ligand **3** (DSGb3α6) at the temperatures of 10 °C (blue circles), 25 °C (green squares) and 35 °C (red diamonds). The solid lines represent the best fit of the experimental data according to a 1:1 binding model equation. All the experiments were performed in PBS buffer, pH 7.4.

The binding affinities of ligands GD3, DSGb3α3, and DSGb3α6 for the protein Siglec‐7 CRD were determined by fluorescence titration experiments (**Figure** **2** and **Table** **1**). Briefly, a protein solution (at fixed concentration of 4 µm) was titrated with a solution of each ligand (Figure S1, Supporting Information). The fluorescence intensity of the protein was then monitored at increasing ligand concentration. We observed a concentration‐dependent reduction in fluorescence intensity of the protein upon ganglioside binding, i.e., the binding of sialoglycans caused a quenching of the emission of the aromatic residues of the protein. The strength of complex formation, quantitatively described by the binding constant (*K*_b_), were determined by non‐linear regression analysis of the experimental data using a 1:1 binding model equation (see Experimental Section).^[^ ^26^ ^]^ In Figure 2, the obtained binding isotherms are shown and the values of the binding constants at the temperature of 25 °C are collected in Table 1.

**Table 1:** Binding constants (*K*_b_) and corresponding equilibrium constants (K_D_) for the complex formation between Siglec‐7 CRD and ligands GD3, DSGb3α3 and DSGb3α6 obtained by means of fluorescence spectroscopy titrations at the temperature of 10, 25, and 35 °C.

Siglec‐7 + ligand 1 (GD3)			Siglec‐7+ ligand 2 (DSGb3α3)			Siglec‐7 + ligand 3 (DSGb3α6)		
T [°C]	K_b_ [M^−1^]	K_D_ [M]	T [°C]	K_b_ [M^−1^]	K_D_ [M]	T [°C]	K_b_ [M^−1^]	K_D_ [M]
10	(1.9±0.7)·10^5^	5.2·10^−6^	10	(8.1±0.2)·10^3^	1.2·10^−4^	10	(1.5±0.6)·10^4^	6.7·10^−5^
25	(6.3±0.1)·10^4^	1.6·10^−5^	25	(2.7±0.2)·10^3^	3.7·10^−4^	25	(1.6±0.4)·10^3^	6.2·10^−4^
35	(4.1±0.1)·10^4^	2.4·10^−5^	35	(1.4±0.3)·10^3^	7.1·10^−4^	35	(1.3±0.4)·10^3^	7.7·10^−4^

An inspection of Table 1 revealed that, at 25 °C, ligand GD3 has the highest affinity for Siglec‐7 CRD, with a *K*_b_ of (6.3 ± 0.1)·10^4^ M^−1^. Instead, for both ligands DSGb3α3 and DSGb3α6, a significantly lower *K*_b_ value was observed (*K*_b_ ≈ 10^3^ M^−1^), indicating a reduced affinity of both.

To understand the possible reasons behind such observations, we determined the thermodynamics parameters of the complex formation by evaluating the temperature dependence of the binding constant and analyzing the data by means of the van't Hoff equation (see Experimental Section). In Figure S2A (Supporting Information), the ln(*K*_b_) versus *T* ^−1^ plots are shown and the *K*_b_ obtained at the explored temperatures are collected in Table S1 (Supporting Information). As observed from the thermodynamics parameters (Table 1), for ligand GD3, an enthalpy change of binding (Δ_b_ *H*°) of −43.6 ± 8.1 kJ mol^−1^ was calculated. To test the reliability of this estimated Δ_b_ *H*° value, we performed a complementary ITC experiment by titrating a solution of the ligand with a solution of Siglec‐7 CRD (see Experimental Section). Since the binding constant was quite low, the ITC experiment couldn't be performed in the usual way, reaching the saturation.^[^ ^27^ ^]^ Instead, the experiment was carried out by injecting a small amount of the protein into the calorimetric cell containing a large excess of the ligand, ensuring that all the injected protein was bound to the ligand. In these conditions, a series of similar heat peaks were obtained (Figure S2B, Supporting Information). The recorded heats, normalized by the moles of injected protein, provided the enthalpy change of binding (Δ_b_ *H*°) whose estimated value is −48.9 ± 3.9 kJ mol^−1^, in excellent agreement with the one obtained by means of the van't Hoff analysis. By means of van't Hoff analysis, the Δ_b_ *H*° values were also determined for ligands DSGb3α3 and DSGb3α6. We found that the Δ_b_ *H*° are −51.1 ± 0.4 kJ mol^−1^ and −63.5 ± 24.4 kJ mol^−1^ for ligand DSGb3α3 and DSGb3α6, respectively. These results clearly indicated that Δ_b_ *H*° was very similar for the three ligands, suggesting that similar interactions were involved (see NMR section below). Of note, it was found that the entropy change of binding (Δ_b_ *S*°) was negative for all the ligands, highlighting that the complex formation was enthalpically driven but entropically disfavored. However, significant differences in the Δ_b_ *S*° values among the ligands were observed, being the lowest in the absolute value for ligand 1 compared to the Δ_b_ *S*° observed for ligand DSGb3α3 and DSGb3α6, revealing that the cause of the reduction of the binding affinity arises from the more negative entropic contribution to the binding leading to a lower Gibbs energy change of binding (Δ_b_ *G°*).

### 2.3 Molecular Binding Between Siglec‐7 and Ligand GD3

**Figure 3:**
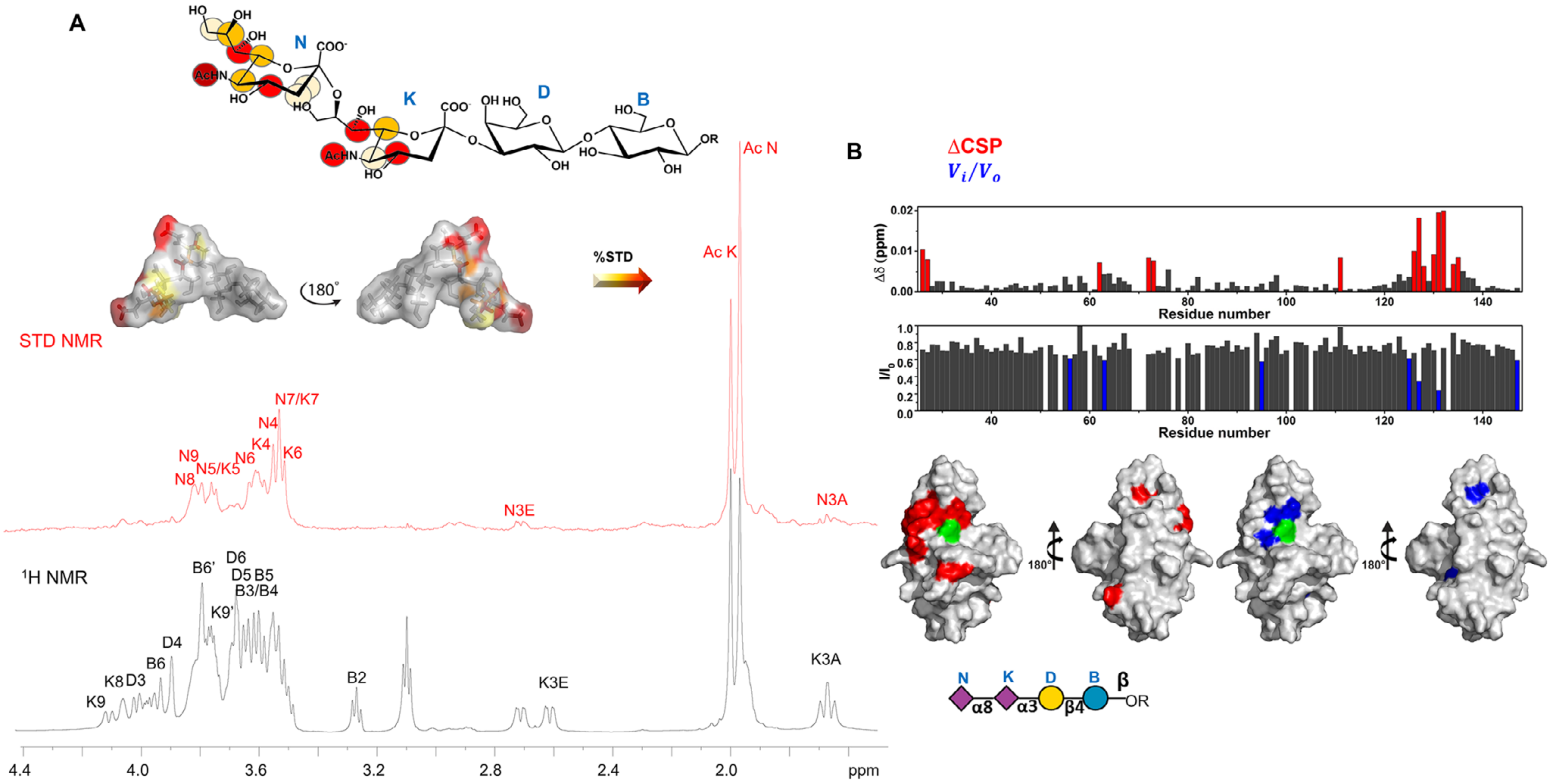
Ligand‐ and Protein‐based NMR analysis of ligand GD3 in interaction with Siglec‐7. A) Epitope mapping of ligand GD3 highlights the protons most involved in interaction with Siglec‐7. %STD values are obtained by the ratio (I_0_ − I_sat_)/I_0_, where (I_0_ − I_sat_) is the signal intensity in the STD‐NMR spectrum (red) and I_0_ is the peak intensity of the off‐resonance spectrum (black). Despite some isochronous signals between N and K, the isolated signals from K, such as K8, as well as the diastereotopic K9 and K3 (axial and equatorial) protons were not recognized by the protein. However, an STD enhancement referring to the acetyl group (AcK) was observed, meaning a partial contribution of the internal sialic acid (K) occurred. Bioactive conformation of ligand GD3, the surface was colored according to the STD effects. B) Plots representing chemical shift perturbation (CSP) in red and decreases in signal intensity in blue. Surface representation of a model of Siglec‐7 with the residues experiencing the largest decrease in intensity in blue and CSP in red in the presence of 200 µm ligand GD3. Arg124 is colored green.

The interaction between Siglec‐7 and GD3 was used to assess the functionality of the recombinant protein, to describe the binding mode, and dissect the 3D complex. The molecular details of the interaction were unveiled by both ligand‐ and protein‐based NMR spectroscopy, as well as computational approaches, including MM and MD simulations (**Figure** **3**).^[^ ^28^ ^]^ Saturation Transfer Difference (STD) NMR experiments were performed on the mixture to map the recognized epitope of GD3, and to determine the protons closest to the receptor surface (Figure 3A). The different profiles between the reference and the STD NMR spectra (in black and red respectively, Figure 3A) clearly indicated a selective binding between Siglec‐7 and ligand GD3. Most STD NMR responses derived from the two sialic acid residues, the main belonging to the terminal sialic acid **N,** indicating its primary involvement in the interaction with Siglec‐7. The highest STD signal originated from the acetyl group of Sia **N** (AcN) and was set to 100%; the other STD effects were derived accordingly. In turn, residue **K** contributed mainly with H7, H4, and its acetyl group, and at lower extent with H5‐H6.

**Figure 4:**
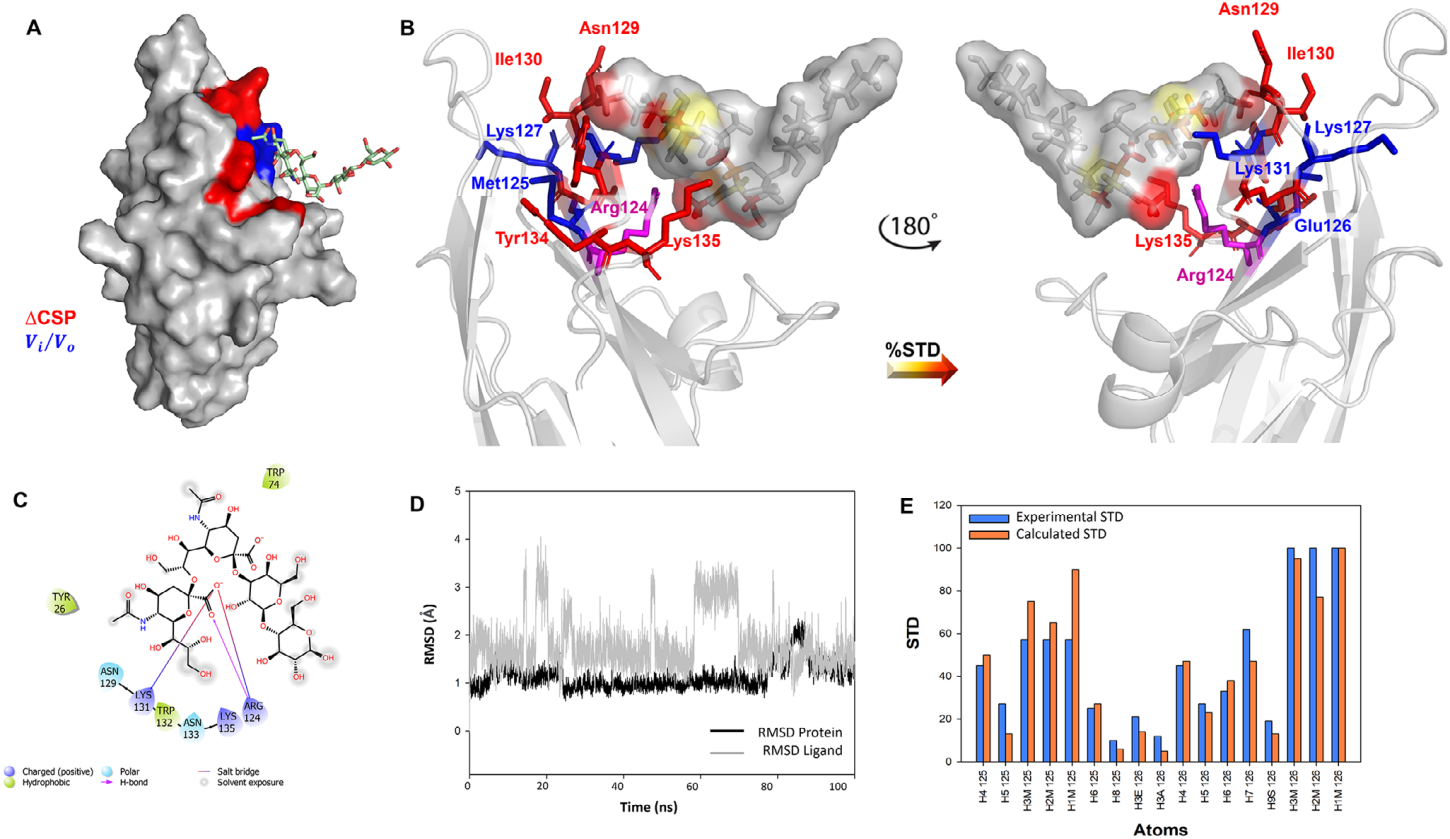
3D model of Siglec‐7CRD ‐ligand GD3 complex. A) 3D complex with the protein surface colored according to the chemical shift perturbation (in red) and intensity decreased (in blue) detected by protein‐based NMR titration. B) 3D views of the Siglec‐7−GD3 complex: the amino acids involved in the interactions as revealed by protein‐based NMR experiments were represented as sticks; the ligand surface was colored according to the STD edit code. C) 2D plot of the interactions occurring at the protein‐ligand interface. D) RMSD along MD simulation. E) Comparison between experimental (blue) and calculated %STD for protons of ligand **1** by RedMat analysis. A NOE R‐factor of 0.2 was calculated.

The interaction of Siglec‐7 with GD3 was further investigated by protein‐based NMR experiments (Figure 3B). Aliquots of GD3 were sequentially added to [U‐^15^N] labeled Siglec‐7 CRD and ^1^H‐^15^N‐HSQC NMR spectra were acquired. As expected, during the NMR titration, some protein signals experienced a decrease in signal intensity and/or chemical shift perturbations (CSP) (Figures 3B and **4**A,B). These changes were significant for cross‐peaks assigned to residues located near Arg124, the canonical residue in the Siglec‐7 binding site. In particular, the signals affected by the largest CSP were assigned to Lys127, Lys131, and Trp132, followed by Glu126, Gly128, Ile130, as well as Tyr134 and Lys135 of GG’ loop, supporting the anticipated interaction involving Arg124. Other CSP effects were observed for Tyr26, Ser27, Arg111, and for His62 of BC loop and Ile72, Ser73 of the CC’ loop. On the other hand, the signals affected by the largest decreases in intensity were assigned to Asn133, which even disappeared with the addition of the ligand, and Lys127 and Lys131, the same residues which further experienced CSP. To a lesser extent, other intensity decreases were also observed for Thr56, Gly63, Phe95, and Met125.

**Figure 5:**
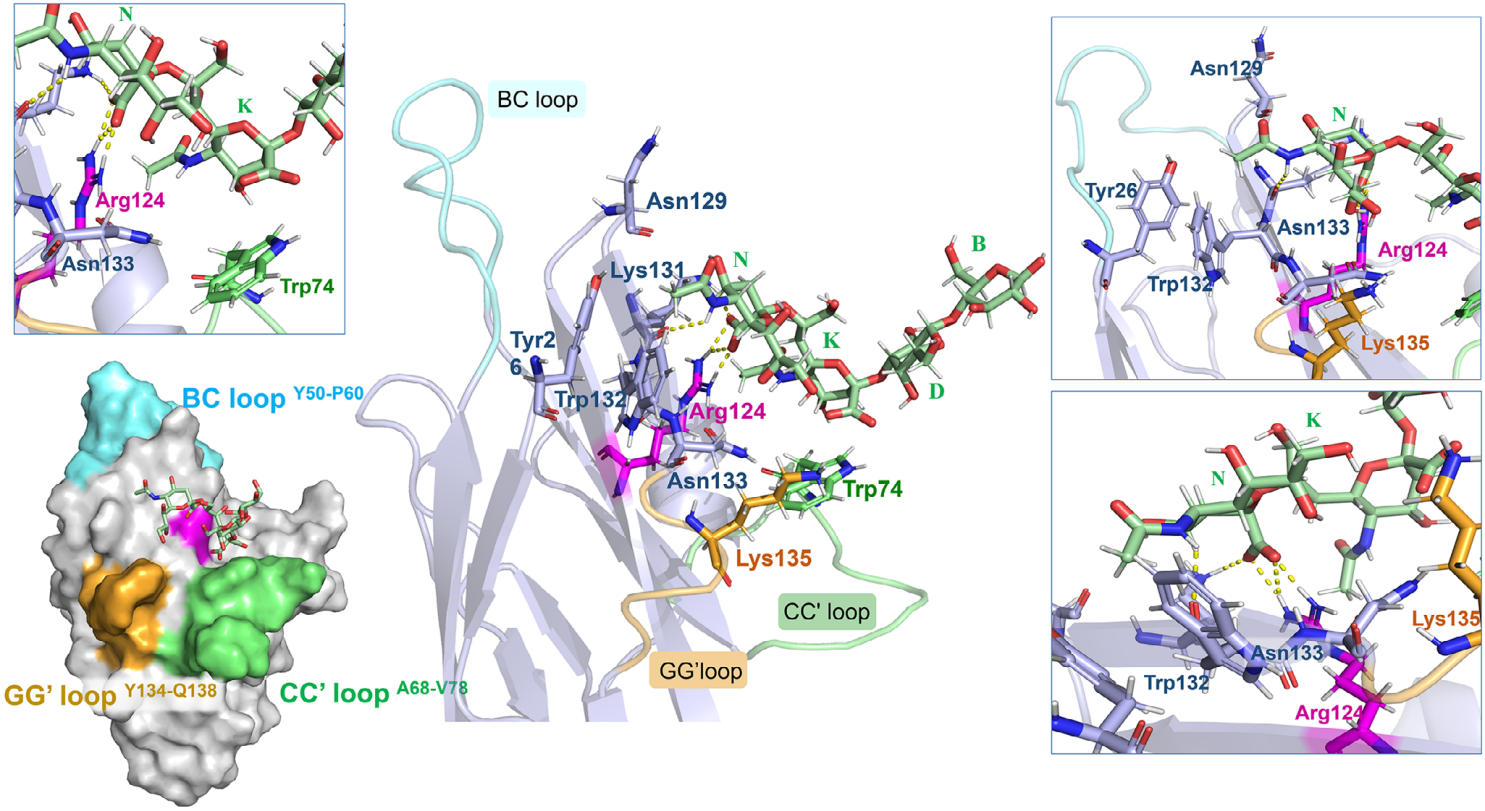
Representation of the Siglec‐7−GD3 complex with the BC, CC’ and GG’ loops highlighted in cyan, green, and orange, respectively. Different views highlighting the H‐bonds monitored by MD simulation were shown (in the zoom, the amino acids were colored according to the loops.

The findings from both NMR titration and STD experiments were essential in constructing the binding site (Figure 4A,B). The α2‐8 linkage connecting the two sialic acids of GD3 shows high levels of conformational flexibility due to the five torsion angles by which it is characterized:  ϕ (C1‐C2‐O‐C8′), ψ (C2‐O‐C8′‐C7′), ω_9_ (O9′‐ C9′‐C8′‐O), ω_8_ (O8′‐C8′‐C7′‐O7′), and ω_7_ (O7′‐C7′‐C6′‐O6′). Once the conformational behavior of GD3 in the free state, previously evaluated,^[^ ^29^ ^]^ was further studied via MD simulations (Figure S3A, Supporting Information), the ligand in its representative pose was modeled into Siglec‐7. Depending on the φ (C1‐C2‐O‐C’3) torsion angle around the Neu5Ac‐α‐(2,3)‐Gal glycosidic linkage,^[^ ^30^ ^]^ ligand 1 could populate two different conformations, namely *–g* and *t*, corresponding to φ values of ‐60° and 180°, respectively. In the bound state, a preference for φ = −60° was observed from MD simulations (Figure S3B, Supporting Information), also supported by tr‐NOESY experiments (Figure S3C, Supporting Information). Overall, the spectral shifts observed in Siglec‐7 upon the addition of ligand 1 were in agreement with a protein‐ligand binding affinity at a low micromolar level. The combination of ligand‐ and protein‐based NMR experiments allowed the modeling of 1 into the binding site. The complex was subjected to MD simulation (Figure S3B, Supporting Information) and the 3D models were evaluated and validated with RedMat^[^ ^24^ ^]^ (Figure 4E), a program that calculates the predicted STD‐NMR intensities from MD trajectories and compares them to the measured STD effects. Notably, the calculated STDs matched well with those experimentally observed, confirming the reliability of the MD simulation on Siglec‐7 and GD3 (ligand 1) and thus of the proposed 3D model in Figure 4. As shown in Figures 4C and **5**, the carboxylate group of the terminal sialic acid established the key salt bridge with the Arg124. Also, a significant H‐bond was detected between the H5N of terminal Sia **N** and Lys131, one of the amino acids most perturbed by both CSP and intensity decrease (Figures 3B and 4A,B). To a lesser extent, H‐bonds between O8 of the glycerol chain of **N** with Asn133 were observed, which was the amino acid mostly perturbed by a decrease in signal intensity (Table S2, Supporting Information).

Regarding the internal Sia residue **K**, an H‐bond between O4 of sialic acid and Trp74 was recurrent during the simulation. The analysis also revealed the presence of hydrophobic amino acids in the binding pocket involving the aromatic components of Trp132 and Trp74, the latter situated within the CC' loop of the protein, in proximity to the residues **N** and **K**, respectively (Figure 4C). In particular, in our 3D model, Trp132 established a π–π interaction with Tyr26, both amino acids affected by CSP variations during the protein NMR titration (Figures 3B and 4B; Table S2, Supporting Information).

Overall, the 3D model of Siglec‐7 and GD3 (ligand 1) proposed here matched NMR binding studies, indicating the terminal sialic acid of the ganglioside as the primary residue involved in the interaction with Siglec‐7 and the internal sialic acid partially contributing to the recognition, while the other residues were solvent exposed.

### 2.4 Molecular Binding Between Siglec‐7 and Ligand DSGb3α3

The molecular recognition of DSGb3α3 by Siglec‐7 was analogously investigated by NMR and computational studies.

**Figure 6:**
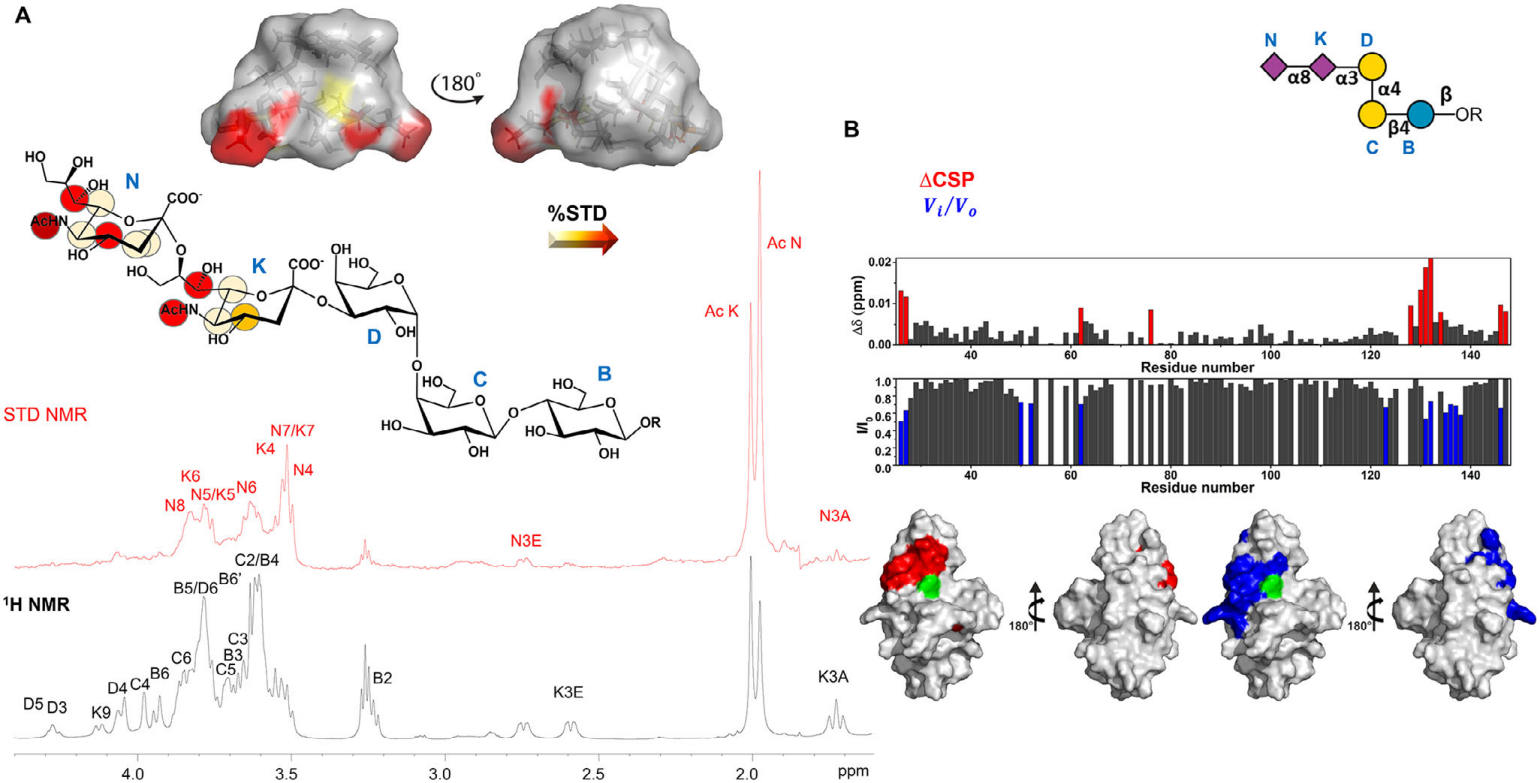
Ligand‐ and Protein‐based NMR analysis of ligand DSGb3α3 in interaction with Siglec‐7. A) Epitope mapping of ligand DSGb3α3 highlights the protons more involved in interaction with Siglec‐7. %STD values are obtained by the ratio (I_0_ − I_sat_)/I_0_, where (I_0_ − I_sat_) is the signal intensity in the STD‐NMR spectrum (red) and I_0_ is the peak intensity of the off‐resonance spectrum (black). Bioactive conformation of ligand DSGb3α3, the surface was colored according to the STD effects. B) Plots representing chemical shift perturbation (CSP) in red and decreases in signal intensity in blue. Surface representation of a model of Siglec‐7 with the residues experiencing the largest decrease in intensity in blue and CSP in red in the presence of 200 µm of ligand DSGb3α3. Arg124 is colored green.

STD NMR experiments indicated that Siglec‐7 mainly accommodates sialic acid residues, primarily the terminal Sia (**N**) (**Figure** **6**A). An inspection of the STD spectra highlighted strong involvement of the acetyl group, H7 and H4 of Sia **N** and Sia **K**, that contributed to a lesser extent. Furthermore, the other sugar residues were mostly excluded from recognition by Siglec‐7 (Figure 6A).

**Figure 7:**
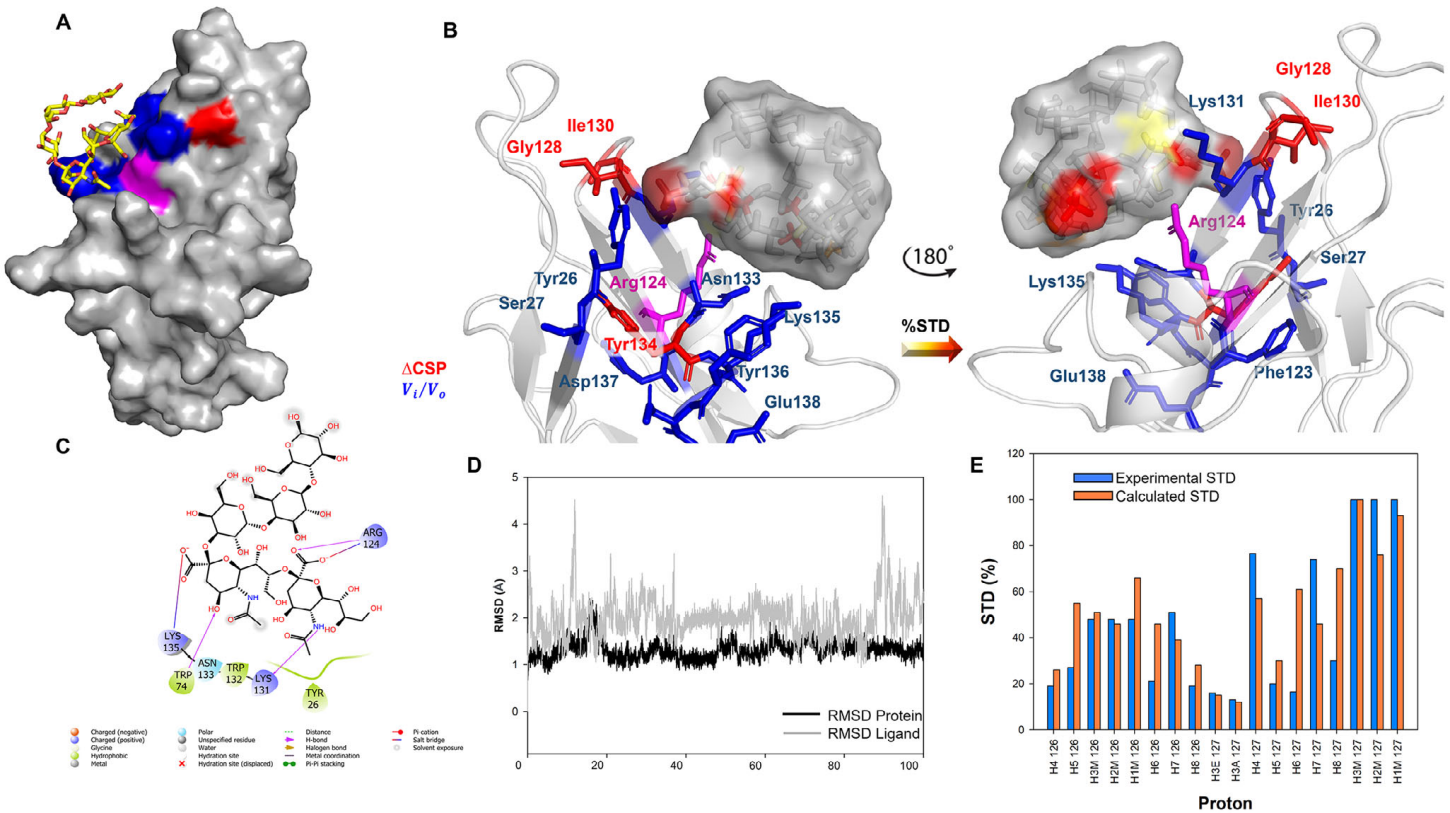
3D model of Siglec‐7CRD ‐ligand DSGb3α3 complex. A) 3D complex with the protein surface colored according to the chemical shift perturbation (in red) and intensity decreases (in blue) detected by protein‐based NMR titration. B) 3D views of the Siglec‐7− DSGb3α3 complex: the amino acids involved in the interactions as revealed by protein‐based NMR experiments were represented as sticks; the ligand surface was colored according to the STD edit code. C) 2D plot of the interactions establishing at the protein‐ligand interface. D) RMSD along MD simulation. E) Comparison between experimental (blue) and calculated %STD for protons of ligand DSGb3α3 by RedMat analysis. A NOE R‐factor of 0.3 was calculated.

Siglec‐7 titration with ligand DSGb3α3 was then performed to have deeper insights into the 3D complex; both CSP and decreases in signal intensity were observed and allowed us to map the region of the protein involved in the interaction with DSGb3α3, especially surrounding the key residue Arg124 (Figures 6B and **7**). In this region, the most significant CSPs were attributed to Gly128, Ile130, Lys131, Trp132, and Tyr134, while Lys131, Trp132, Asn133, and all the amino acids from Lys135 to Gln138 of the GG’ loop experienced a decrease in signal intensity. The residues of GG’ loop (Tyr134 by CSP and Lys135, Tyr136, Asp137, and Glu138 by intensity decreases) were indeed particularly influenced by the presence of ligand DSGb3α3; we also observed a significant CSP for the signal of Ala76 of the BC loop and decreases in signal intensity for residues belonging to the BC loop (Tyr50 and Val52). Besides Lys131, and Trp132, which were affected by both kinds of changes, we also found Tyr26, Ser27, and His62, close in space to the binding site experiencing CSPs (Figure 7). The bioactive conformation adopted by ligand **2** upon binding to Siglec‐7 was then explored by tr‐NOESY experiments coupled to MD simulation.

In the free state, ligand DSGb3α3 could populate *–g* and *t* conformations according to the φ (C1‐C2‐O‐C’3) torsion angle around the Neu5Ac‐α‐(2,3)‐Gal glycosidic linkage (−60° and 180° respectively, Figure S4, Supporting Information). Therefore, an MD simulation in the bound state was first carried out with ligand DSGb3α3 in the *t* conformation (Figure S5A, Supporting Information), maintained for approximately 20 ns after which the φ torsion angle turned to ‐60° (change from *t* to *‐g*), maintained until the end of the MD simulation. In addition, a second MD simulation in the bound state was undertaken considering ligand DSGb3α3 in *‐g* conformation as the starting pose (Figure S5B, Supporting Information): for the entire simulation time, the φ torsion angle populated the value of −60°. Therefore, in both MD simulations of the bound states, the preferred conformation of the φ torsion angle around Neu5Ac‐α‐(2,3)‐Gal of ligand DSGb3α3 appeared to be −60°.

Confirmation came from a detailed analysis of key tr‐NOE contacts, that indicated a propensity of DSGb3α3 for adopting the *‐g* conformation, supported by a decrease in NOE intensity corresponding to K3_ax_‐D3, and by the appearance of a weak NOE corresponding to K9‐D3. Regarding the torsion angles around Neu5Ac‐α‐(2,8)‐Neu5Ac linkage, φ (C1‐C2‐O8‐C8) can populate different conformational states between −60° and −180° in the free state (Figure S4, Supporting Information), while φ at −60° was favored in the bound state (Figure S5B, Supporting Information). In addition, tr‐NOE contacts between the terminal sialic acid (**N**) and the glucose residue (**B**) further suggested that ligand DSGb3α3 adopted a bent conformation upon binding to Siglec‐7, matching with a φ torsion angle around Neu5Ac‐α‐(2,8)‐Neu5Ac at −60° (Figure 7).

**Figure 8:**
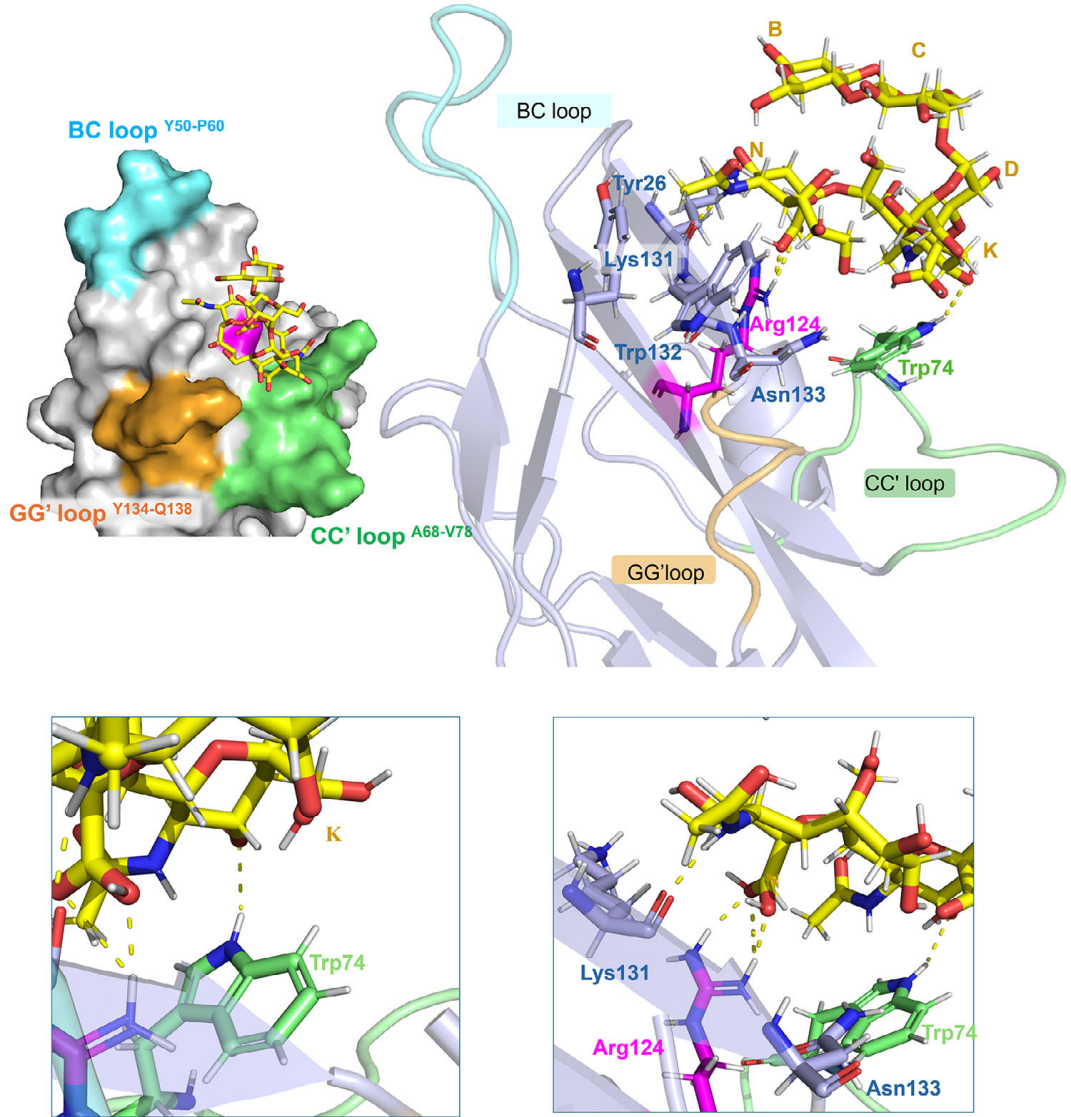
Representation of the Siglec‐7− DSGb3α3 complex with the BC, CC’ and GG’ loops highlighted in cyan, green, and orange, respectively. Different views highlighting the H‐bonds monitored by MD simulation were shown (in the zoom, the amino acids were colored according to the loops legend).

Notably, these torsions remained stable along the MD simulation (Figure S5, Supporting Information). Moreover, the MD trajectories were analyzed through RedMat algorithm (Figure 7E) and a 3D model of Siglec‐7 with DSGb3α3 was proposed (**Figure** **8**).

The results illustrate the critical role of the carboxylate group of the terminal sialic acid **N** in establishing a salt bridge with Arg124. Additionally, hydrogen bonds were identified again between the H5N of the terminal sialic acid **N** and Lys131, affected by both CSP and decrease of signal intensity, as well the previous interactions with Asn133, which showed a substantial reduction in signal intensity during the protein NMR titration (Figure 6B). As for the internal Sia residue **K,** this residue was facing toward the aromaticTrp74 belonging to the CC’ loop by which a hydrogen bond was also established (Figures 7C and 8). Other aromatic amino acids located in the binding site included Tyr26 and Trp132, which were affected by both CSP and intensity decreases and established π‐π interactions with each other.

### 2.5 Molecular Binding Between Siglec‐7 and Ligand DSGb3α6

**Figure 9:**
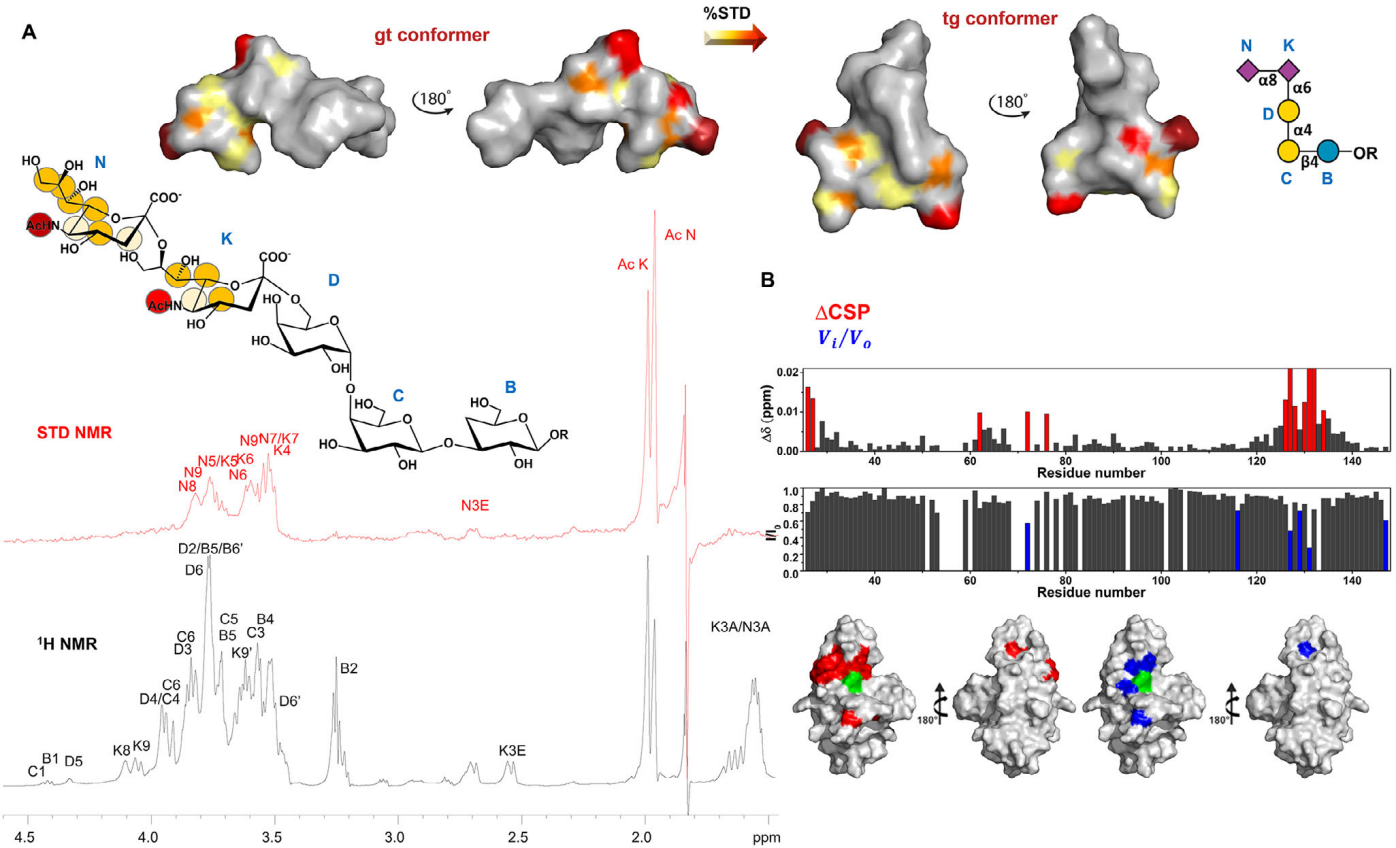
Ligand‐ and Protein‐based NMR analysis of ligand DSGb3α6 in interaction with Siglec‐7. A) Epitope mapping of ligand **3** highlights the protons more involved in interaction with Siglec‐7. %STD values are obtained by the ratio (I_0_ − I_sat_)/I_0_, where (I_0_ − I_sat_) is the signal intensity in the STD‐NMR spectrum (red) and I_0_ is the peak intensity of the off‐resonance spectrum (black). Bioactive conformation of ligand DSGb3α6, the surface was colored according to the STD effects. B) Plots representing chemical shift perturbation (CSP) in red and decreases in signal intensity in blue. Surface representation of a model of Siglec‐7 with the residues experiencing the largest decrease in intensity in blue and CSP in red in the presence of 200 µm ligand DSGb3α6. Arg124 is colored green.

NMR and computational studies have also been performed to study the molecular recognition of ligand DSGb3α6 by Siglec‐7. Similarly to ligand DSGb3α3, STD NMR experiments of ligand DSGb3α6 showed that the terminal Sia residue (**N**) was crucial for the interaction (**Figure** **9**A) and contributed from N4 to N9, including the acetyl group that gave the 100% STD effect.

**Figure 10:**
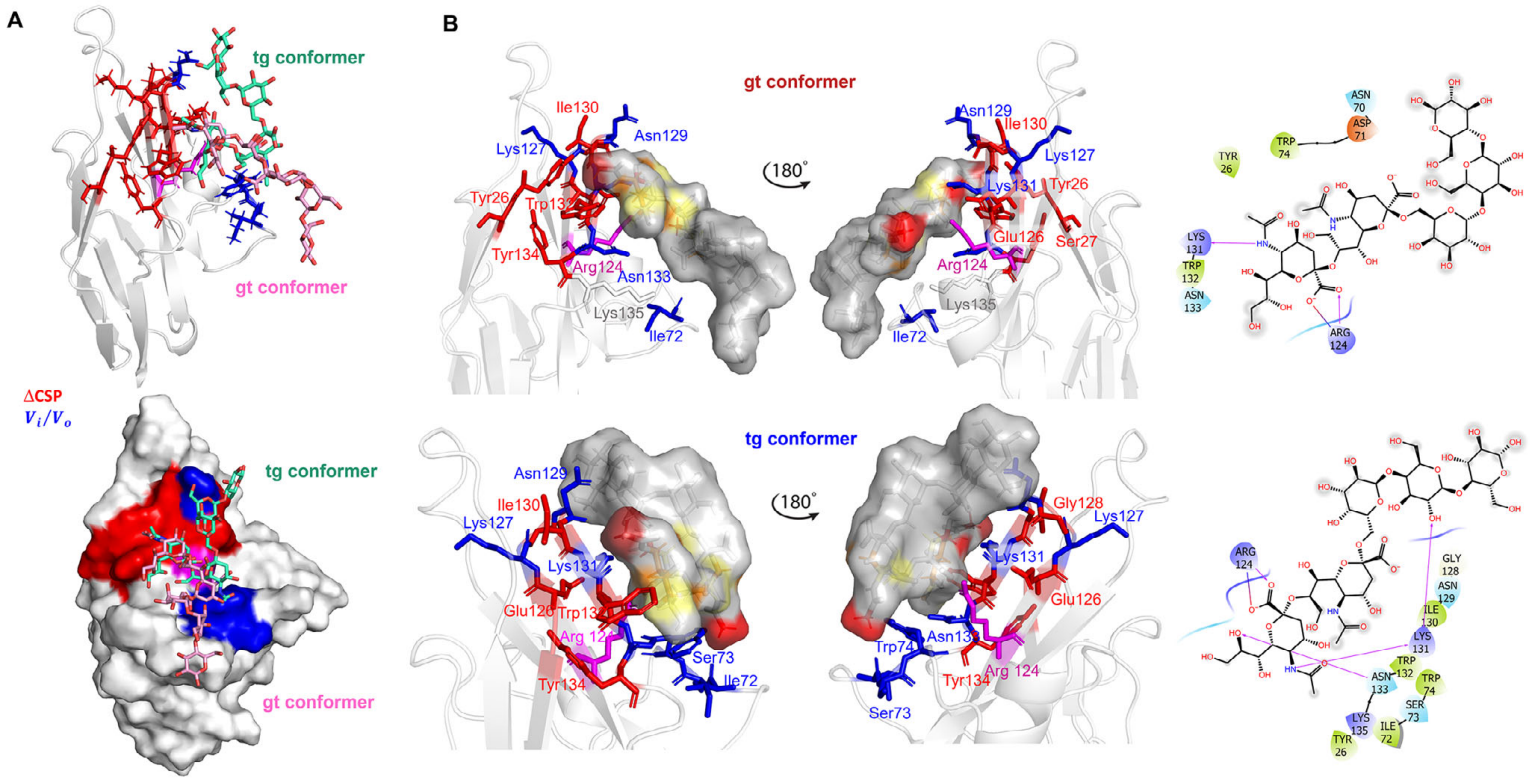
A) Superimposition of 3D models of Siglec‐7CRD bound to ligand DSGb3α6 in *tg* and *gt*. B) 3D views of the Siglec‐7− DSGb3α6 complex: the amino acids involved in the interactions as revealed by protein‐based NMR experiments were represented as sticks; the ligand surface was colored according to the STD edit code. 2D diagram of the interactions established at the protein‐ligand interface, with DSGb3α6 in *tg* (A) and *gt* (B) conformations.

Interestingly, as for the diastereotopic methylene signals of Sia, a low STD enhancement was only noticed for N3E. Regarding Sia **K**, medium to low STD responses were attributed to K4, K7, K6, and the acetyl group. Similarly to ligands GD3 and DSGb3α3, the other residues were excluded from interaction with Siglec‐7, as indicated by the absence of corresponding STD NMR signals. The molecular binding between Siglec‐7 and ligand DSGb3α6 was also investigated by protein‐based NMR binding experiments (Figure 9B). Both CSP and decreases in signal intensity were observed during the titrations, which allowed the definition of the binding site of Siglec‐7 (**Figure** **10**). Therefore, residues from Glu126 to Tyr134, in the vicinity of Arg124, exhibited consistent perturbations; in particular, Lys127, Lys131 and Trp132 experienced the largest CSP, followed by Glu126, Gly128, Ile130, and Tyr134; the remaining amino acids were instead affected by intensity decrease (Lys127, Asn129, Lys131 and Asn133). Notably, Lys127 and Lys131 were affected by both CSP and intensity decrease. Moreover, the amino acids in proximity to this latter region included Tyr26, and Ser27, which only gave CSP effects. On the other hand, lower perturbations were evidenced for residues His62 of the BC loop and Ala76 of the CC loop influenced by CSP and for Ile72 of the CC loop affected by both CSP and intensity decrease (Figures 9B and 10).

**Figure 11:**
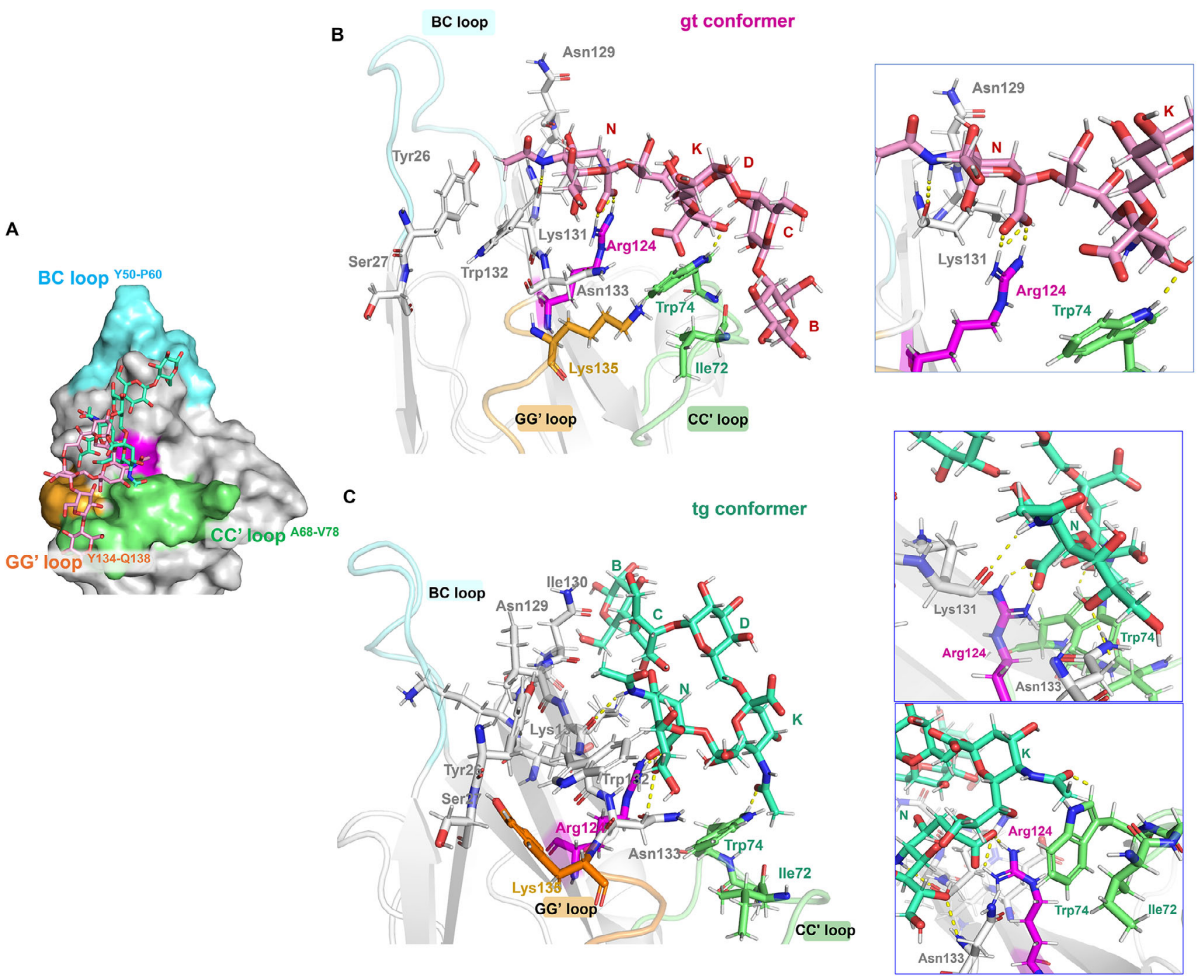
Representation of the Siglec‐7− DSGb3α6 complex in both *gt* (pink) and *tg* (cyan) conformations with the BC, CC’, and GG’ loops highlighted in cyan, green and orange, respectively. A) Different views of the Siglec‐7− DSGb3α6 complex in the *gt* conformation highlighting the H‐bonds monitored by MD simulation were shown (in the zoom, the amino acids were colored according to the loops legend). B) Different views of the Siglec‐7− DSGb3α6 complex in the *tg* conformation highlighting the H‐bonds monitored by MD simulation were shown (in the zoom, the amino acids were colored according to the loops legend).

Tr‐NOESY experiments and computational studies allowed us to depict the 3D model. Thus, Neu5Acα2‐6‐Gal linkage was affected not only by φ (C1‐C2‐OC6′) and ψ (C2‐O‐C6′‐C5′), but by an additional ω torsion angle around the C5′‐C6′ (O‐C6′‐C5′‐O5′). Previous computational studies in the free state (Figure S7A, Supporting Information) showed the ψ angle mainly acquired values around 180°^[^ ^31^ ^]^ also maintained in the bound state (Figure S7B, Supporting Information). As for the φ torsion angle, the absence of NOE contacts between the diastereotopic protons at position 3 of sialic acid (K3A and K3E) and protons at position 6 of galactose (D residue) is an indication of a preference for the ‐g conformation (Figure S8A, Supporting Information), in turn confirmed by MD simulations (Figure S7, Supporting Information). Additionally, the ω torsion angle could populate three different values in the free state, 60°/180°/−60°, corresponding to *gt/tg/gg* conformations, respectively.^[^ ^26^, ^32^ ^]^ Overall, both MD simulations (Figure S7A,B, Supporting Information) and tr‐NOESY NMR experiments (Figure S8A, Supporting Information) revealed no conformational differences between free and bound state, and that both *tg* and *gt* conformations were likely populated (Figure 10). Indeed, ω at −60° was less energetically favored and the different multiplicity of the diastereotopic protons at position 6 of the galactose residue D observed on the ^1^H^13^C‐HSQC spectrum confirmed the exclusion of the *gg* conformation (Figure S8B, Supporting Information). As for the other two torsions, no significant NOE contacts were found for ligand DSGb3α6 to discriminate in the bound state between the two conformers (Figure S7B, Supporting Information). The coexistence of both *gt* and *tg* conformers was further supported by protein‐based NMR experiments (**Figure** **11**), which showed that amino acids located in the CC' loop of the protein, such as Ile72 and Trp74, were affected in both cases. This also occurred with amino acids nearby or part of the GG' loop such as Lys131, Trp132, Asn133, Tyr134, or Lys135. The interaction of the key Arg124 with the terminal sialic acid **K** of the ligand occurred in both *gt* and *tg* conformations, hydrogen bonds could also be identified between Lys131 and the terminal sialic acid, in particular the NH of the acetyl group. Particularly, in the *tg* conformation the H‐bonds between the terminal sialic acid (**N**) O8 and (at lesser extent) O9 of the glycerol chain and Asn133 were also observed. Within the internal Sia **K** residue, a hydrogen bond with Trp74 was observed in both conformers (Figure 11 and Table S2, Supporting Information).

Therefore, the combination of ligand‐based and protein‐based NMR with MD simulations and Redmat calculations allowed us to obtain 3D models of the Siglec‐7‐ganglioside complexes, highlighting the interactions occurring at the interface. To investigate whether the glycosidic linkages of gangliosides could affect their binding accommodation, apart from the main ganglioside GD3 we further evaluated the molecular binding with modified GD3 structures. Therefore, we used two Gb3 derivatives, DSGb3α3 and DSGb3α6, which maintained the disialylated Neu5Ac‐α‐(2‐8)‐Neu5Ac portion and varied in the linkage of the internal sialic acid to galactose and in length of the glycan chain We found that the *K*_b_ values follow the order GD3 > DSGb3α3 ≈ DSGb3α6, thus GD3 has the highest affinity for the protein (Table 1). To disentangle the intricate observed behavior, we evaluated the thermodynamics parameters (namely Δ_b_ *H°* and Δ_b_ *S°*) of the interaction by determining the temperature‐dependence of *K*_b_. We found that the Δ_b_ *H°* is negative for all the ligands highlighting the formation of non‐covalent interactions (as H‐bonds, salt bridges, Table S2, Supporting Information) between the residues in the binding pocket of the protein and the sugars. Interestingly, the Δ_b_ *H°* values are very similar for all the ligands, indicating the enthalpy contribution to the binding Gibbs energy is not responsible for the observed alteration in the binding constant. Indeed, the sialic acid moieties of GD3, DSGb3α3, and DSGb3α6 are the only residues involved in the interaction with Siglec‐7, with a bias for the terminal Sia of the Neu5Ac‐α‐(2‐8)‐Neu5Ac glycosidic linkage, while the other sugars result solvent exposed and, consequently, represent the more flexible portions of the ligands. On the other hand, an inspection of Table S1 (Supporting Information) highlights significant differences among the three ligands in the entropy change of binding (Δ_b_ *S°*). Of note, the Δ_b_ *S°* is more negative for ligands DSGb3α3 and DSGb3α6 with respect to ligand GD3 indicating that the entropy penalty to be paid for complex formation is higher for ligands DSGb3α3 and DSGb3α6. Several reasons can be invoked for such observations. However, our NMR and computational results pointed out how ligands DSGb3α3 and DSGb3α6 possess higher rotational degrees of freedom in the unbound state compared to ligand GD3. Consequentially, upon complex formation, the reduction of such degrees of freedom is more prominent for ligands DSGb3α3 and DSGb3α6, leading to more negative Δ_b_ *S°*, which in turn, causes an increase of Δ_b_ *G°*, disfavoring the complex formation. Notably, the most negative entropy was observed for the complex with ligand DSGb3α6, which indeed shows a conformational equilibrium between *tg* and *gt* rotamers in the bound state.

Furthermore, protein‐based NMR experiments could profile the binding site of Siglec‐7. In all complexes the amino acids mostly affected by the gangliosides corresponded to those located in the proximity of Arg124, crucial for sialoglycan binding since it establishes the key salt bridge with the carboxylate group of the terminal Sia. For example, Gly128, as well as the region close to and partially including the GG’ loop, the residues from Ile130 to Tyr134^GG’^, were affected by the largest CSP (Gly128, Ile130, Lys131, Trp132^GG’^, and Tyr134^GG’^) and decrease of signal intensity (Lys131 and Asn133^GG’^) in all the three complexes. Additionally, other residues (Tyr26, Ser27, and His62^BC^) structurally close to the binding region were found to be affected by significant CSPs in the presence of all the three ligands. These results support the importance of the binding site neighboring Arg124, which always establishes a key salt bridge with the carboxylate group of the terminal sialic acid. In particular, the terminal Sia of all gangliosides establishes hydrogen bonds mainly through its carboxylate group, NH of the acetyl group and the glycerol chain with the arginine sidechain. Interestingly, in the structural models of the complexes, we also observed this residue in close contact with the aromatic residue Trp132^GG’^, further stabilized by π–π interactions with Tyr26. This model is backed by the NMR data, since both aromatic residues experience significant CSPs in the presence of all the three ligands. Moreover, two different lysine residues in the binding site of Siglec‐7 are also perturbed in the NMR spectra: a sizable CSP is observed for Lys135^GG’^ in the presence of both GD3 and DSGb3α3, and decrease in signal intensity is detected for Lys127 in the presence of both GD3 and DSGb3α6. Overall, the numerous chemical shift perturbations and the lower effects in signal intensity decrease of the residues in the binding site of Siglec‐7 agree with the binding affinities in the micromolar range calculated by fluorescence analyses. This is also consistent with the binding affinities calculated with other gangliosides; indeed, Siglecs–carbohydrate interactions typically exhibit low affinity, relying on high avidity conferred by multivalency and ligand clustering.^[^ ^33^, ^34^ ^]^ The combination of the results of protein‐based NMR assay and ligand‐based STD experiments is instrumental in delineating binding site characteristics of Siglec‐7, which is found to be influenced by interactions with ligands GD3, DSGb3α3, and DSGb3α6.

## 3 Conclusion

Gangliosides are sialylated glycosphingolipids ubiquitous in all human cells, very abundant in the brain and nervous system. Based on their location, they play several biological roles under healthy conditions, such as cell–cell and cell‐matrix interactions, modulation of signal transduction, and coordination of signal events with other cells. However, ganglioside composition can be altered during malignant transformation and high levels of ganglioside expression contribute to pathological conditions, such as GD3 and GD2 in melanoma and neuroblastoma respectively, leading to tumor formation and progression. Moreover, the presence of gangliosides was detected in the blood of certain cancer patients, making them potential biomarker for tumor diagnosis and/or prognosis or targets for antibody therapies.^[^ ^35^, ^36^ ^]^ In vitro studies have shown that most gangliosides containing one or two sialic acids have suppressive effects against the activation of immune cells, such as B cells, NK cells, dendritic cells, and monocytes, promoting tumor progression. Inhibition of NK cells activity and regulation of NKs cytotoxicity by GM3, GM2, and GD3, also occur through interaction with Siglecs. Indeed, GD3 is strongly engaged by Siglec‐7 on NK cells, leading to immunosuppressive responses in vitro and in vivo.^[^ ^37^ ^]^ Furthermore, dehydroxylated ceramide‐containing GD3 reduced the sensitivity to NK cells, indicating that modifications in ceramide‐containing glycosphingolipids not only influence Siglec‐7 binding properties but also impact the biological effects triggered by their interactions.^[^ ^38^ ^]^ Notably, cancers typically show significantly lower levels of FA2H (fatty acid 2‐hydroxylase) than corresponding normal tissues, such as skin and colon. Consequently, loss of hydroxylation of ceramides in cancers may enhance the ability of cancer cells to evade NK cell attacks, leading to the escape of cancer cells from immune surveillance system.^[^ ^38^ ^]^

Siglec‐7 is an inhibitory immune receptor that is emerging as a significant target of interest for cancer immunotherapy. Siglec‐7 ligands offer valuable insights into motifs and key elements essential for its engagement and the consequent evasion of immune recognition. This interaction establishes Siglec‐7 and corresponding ligands as innovative glyco‐immune checkpoints in the realm of cancer treatment. In this frame, the structure of the N‐terminal V‐set domain of Siglec‐7 interacting with a synthetic analog of GT1b was solved.^[^ ^39^ ^]^ The presence of the branch and the axial position of the Gal C4‐O make GT1b highly restricted, influencing the ligand's conformation and recognition. Despite Neu5Acα(2,8)Neu5Acα glycosidic linkage in GT1b adopts a stereochemical arrangement that varies between antiperiplanar and anticlinal with respect to the synclinal conformation observed in GD3 and Gb3 derivatives studied in this work, the terminal sialic acid remains the major determinant of ligands’ binding in all cases. Additionally, hydrogen bonds involving terminal Neu5Ac‐N5 with Lys131‐CO and terminal Neu5Ac‐O8 with Asn133‐N were consistently observed. However, in GT1b, Neu5Ac‐O9 establishes H‐bonds with Lys135‐NZ and Asn133‐CO, whereas in GD3, the diastereotopic protons of the glycerol chain are more distant from the protein. Sato et al. proposed a model of Siglec‐7 in which two binding sites contribute to glycan recognition: the primary site, where R124 is located, and a secondary site containing other arginine residues, R67 and especially R94, both situated in a cavity placed under the CC’ loop. In our complexes, the salt bridge between the carboxyl group of the non‐reducing terminal Sia and Arg124 remains stable for the entire simulations, confirming it as the primary site, excluding Arg67 and Arg94 from the interaction.^[^ ^40^, ^41^ ^]^

In the context of the Siglecs‐gangliosides axis of modulation of immune response and tumor microenvironment, antibody therapies have been investigated in pre‐clinical and clinical studies^[^ ^42^ ^]^ and synthetic mimetics of carbohydrate portions of GD2 and GD3 gangliosides have been tested as potential vaccines,^[^ ^43^ ^]^ although they only partially succeed. Therefore, the effectiveness of ganglioside mimetic vaccines needs to be improved to be effective. Consequently, molecular‐level understanding of gangliosides interaction with Siglec‐7 can not only shed light on the mechanisms underlying immune evasion in cancer, but also help the development of targeted strategies to modulate these interactions. With this aim, we combined structural biology methodologies, NMR techniques, biophysical studies, and computational approaches to provide 3D models and binding affinities of Siglec‐7 interacting with the carbohydrate moieties of its preferred ganglioside, GD3, and structurally related Gb3 derivatives. Notably, the lipid portion of ceramide can enhance the interaction of gangliosides with Siglec‐7 and may influence the orientation of the C‐C′ loop; however, the underlying molecular mechanisms remain to be investigated.^[^ ^40^ ^]^

By incorporating the unique effects of each ligand on the protein structure and dynamics, this deep understanding of Siglec‐7 interaction with ganglioside and their impact on binding site architecture can be achieved for further optimization and drug development. In this work, we highlighted the crucial role of thermodynamics in explaining the binding affinities of protein‐ligand interactions. Indeed, despite GD3 and Gb3 derivatives show comparable binding epitopes and are recognized by a similar set of amino acids in the primary site of Siglec‐7, the ligands’ conformational behavior influences the formation of the complexes, and thus the affinity of the binding. Indeed, the higher flexibility of Gb3 derivatives, especially DSGb3α6, reflects in a loss of entropy upon binding to Siglec‐7, increasing Δ_b_G° and reducing affinity.

In addition, Siglec‐7′s reported binding to α(2→3) and α(2→6)‐linked sialosides underscores the need for comparative analyses of its interactions with these glycans, following our focus on α(2→8)‐linked gangliosides. A direct comparison of Siglec‐7 binding to all three linkages (α(2→3), α(2→6), and α(2→8)) will be essential for a comprehensive understanding of its ligand specificity. This is particularly important because these linkages exhibit distinct structural presentations and biological roles. Therefore, exploring the structural diversity of gangliosides and their dynamic interplay with Siglec‐7 provides an understanding of the intricate cellular communication processes, offering potential avenues for therapeutic interventions to restore immune recognition and enhance anti‐cancer immune responses.

## 4 Experimental Section

### Expression and Purification of the Full Extracellular Domain (FED) of Siglec‐7 in HEK293 Cells

The Siglec‐7 FED (Gly18‐Gly354) with a C‐terminal histidine tag was expressed in suspension‐adapted human HEK293S GnTI^−^ cells, as previously indicated.^[^ ^44^ ^]^ Briefly, Siglec‐7 FED was produced as a glycoprotein containing oligo‐mannose type glycans to avoid interferences from sialic acids during the binding with the ligands. ExCELL 293 serum‐free cell culture medium (Merck) was used for cell line cultivation and transfection. The protein was purified from the cell culture supernatant by immobilized metal affinity chromatography (IMAC) followed by size‐exclusion chromatography (SEC). The glycosylated Siglec‐7 FED was used for the ligand‐based NMR binding studies.

### Expression and Purification of the Carbohydrate Recognition Domain (CRD) of Siglec‐7 in *E. coli* Cells

The carbohydrate recognition domain (CRD) of Siglec‐7 was cloned to pET29b(+) vector in between NdeI and XhoI restriction sites. The vector sequence was designed as previously published.^[^ ^45^ ^]^ In brief, the coding region containing Gly18‐His148 sequence from the full‐length mature protein was codon optimized by Twist Bioscience. An additional modification to the lectin domain was done by replacing Cys41 with a serine residue, to avoid unspecific disulphide bridge formation.

The Siglec‐7 CRD carrying‐plasmid was transformed into BL21(DE3) Codon Plus RIPL or Rosetta 2 cells with the specific antibiotic selection and cultured at 37 °C, at 180 rpm, in either LB or M9 culture medium, correspondingly supplemented with 1 mm MgSO_4_, 0.3 mm CaCl_2_, 0.2% v/v solution Q, 1 µg mL^−1^ thiamine, 1 µg mL^−1^ biotin, 1 g (^15^NH_4_)_2_SO_4_ and 3 g [U‐^13^C] glucose for isotopic labeling. The overexpression was induced at OD_600_ = 0.8 with 1 mm IPTG (isopropyl β‐D‐1‐thiogalactopyranoside) and incubated further for 24 h at 25 °C under 180 rpm. The cells were harvested at 7500 rpm, for 20 minutes, using a Sorvall RC 6 Plus Superspeed Centrifuge (Thermo Scientific) and then collected and resuspended in the lysis buffer (20 mm potassium phosphate, 500 mm NaCl, 20 mm imidazole, pH 7.4) and subsequently sonicated for 10 cycles (30 seconds ON, 2.5 minutes OFF) at 70% amplitude, using a Vibra‐Cell sonicator (Sonics & Materials). The lysate has been ultracentrifuged for 40 minutes at 40 000 rpm with Optima LE‐80K Ultracentrifuge (Beckman). Siglec‐7 CRD was then found incorporated into inclusion bodies. While supernatant was discarded, the inclusion bodies were resuspended in 8 m urea lysis buffer (20 mm potassium phosphate, 500 mm NaCl, 20 mm imidazole, pH 7.4) and re‐sonicated. The solubilization of the inclusion bodies has been repeated for improved recovery yield of the protein of interest. The soluble, unfolded fraction containing Siglec‐7 CRD has been further subjected to IMAC purification on a HisTrap FF (5 mL, GE Healthcare). The column was previously equilibrated with 20 mm potassium phosphate, 500 mm NaCl, 20 mm imidazole, pH 7.4, and the protein was loaded at a flow rate of 3 mL min^−1^, in closed cycle, for 2 h. The column was eluted at a 4 mL min^−1^ flow rate, with high‐concentration imidazole buffer (20 mm potassium phosphate, 500 mm NaCl, 500 mm imidazole, pH 7.4) (Figure 1A,B). To proceed with the refolding of the unfolded, purified protein of interest, a series of dialysis were set in place. The protein was placed in a 10 kDa cut‐off membrane and each dialysis was performed overnight by changing the buffer in a 2 L tank, as it follows: 1st dialysis) 20 mm potassium phosphate, 300 mm NaCl, 4 m urea; 2nd dialysis) 20 mm potassium phosphate, 300 mm NaCl, 2 m urea; 3rd dialysis) 20 mm potassium phosphate, 150 mm NaCl. Siglec‐7 CRD, now refolded, was collected from the membrane and ultracentrifuged out from the formed aggregates at 40 000 rpm, for 40 minutes and then, filtered on a 0.2 µm micropore membrane. The final purification was achieved by a size‐exclusion chromatography on a HiLoad 26/60 Superdex 75 pg (GE Healthcare) coupled on an AKTA Go FPLC system, equilibrated with 20 mm potassium phosphate, 50 mm NaCl, pH 7.4 (Figure 1C,D). The non‐glycosylated Siglec‐7 CRD was used for protein‐based NMR binding studies.

### Backbone Resonance Assignment of Siglec‐7

NMR protein experiments for backbone assignment were acquired on a sample of [U‐^15^N‐^13^C] Siglec‐7 at the concentration of 400 µm in 600 µL of aqueous buffered solution (20 mm potassium phosphate, pH 7.4, 50 mm NaCl, 0.01% NaN_3_, 1 mm of protease inhibitors, 10% D_2_O, in a 5 mm NMR tube. Triple resonance experiments for protein NMR assignment HNCA, HNCACB, HNCO, and CBCAcoNH were recorded at 298 K on a Bruker's Avance^TM^ NEO 900 MHz spectrometer, equipped with TCI cryo‐probe. 3D HNcaCO was recorded at 298 K on a Bruker's Avance^TM^ 500 MHz spectrometer. 93% of the amino acid sequence from Y26 to T147 was assigned, excluding the 5 proline residues. Data acquisition and processing was performed on TOPSPIN 4.1.1 software and spectra were analyzed by using CARA (Computer Aided Resonance Assignment) software.^[^ ^46^ ^]^

### Protein‐Based NMR Experiments

For ligand binding studies, 2D ^1^H‐^15^N HSQC NMR experiments were recorded on samples of 200 µm [U‐^15^N] Siglec‐7 CRD in 200 µL of aqueous buffered solution (20 mm potassium phosphate, pH 7.4, 50 mm NaCl, 0.01% NaN_3_, 1 mm of protease inhibitors, 10% D_2_O in a 3 mm NMR tube. Experiments were acquired on a Bruker's Avance™ NEO 900 MHz spectrometer, equipped with triple resonance TCI cryo‐probe. The spectra were acquired using 32 scans with 2048 data point in t2, 128 increments in the indirect dimension (t1), a recycle delay of 1.2 seconds, and the temperature was kept at 298 K. The interaction of Siglec‐7 CRDwith the ligands was investigated by adding increasing amounts ligands GD3, DSGb3α3, or DSGb3α6, to three different tubes containing Siglec‐7 CRD, to reach ligand concentrations of 12.5, 25, 50, 100, 200, 400, 800, 1600, and 3200 µm. 2D ^1^H‐^15^N HSQC spectra were acquired after the addition of each ligand aliquot. Data acquisition and processing were performed with TOPSPIN 4.1.1 software and the spectra were analyzed using CARA. Chemical Shift Perturbations (CSP) were evaluated using the equation:^[^ ^47^, ^48^ ^]^

(1) $$\Delta \delta =\frac{1}{2}\;\sqrt{\Delta \delta_{H^{2}}+{\left(\frac{\Delta \delta_{N}}{5}\right)}^{2}}$$

### Ligand‐Based NMR Experiments

Saturation Transfer Difference (STD) and transferred‐NOESY NMR experiments were recorded on a Bruker AVANCE NEO 600‐MHz equipped with a cryo‐probe and data acquisition and processing were performed with TOPSPIN 4.1.1 software. Samples were prepared in phosphate saline deuterated buffer (10 mm Na_2_HPO_4_, 2.7 mm KCl, 137 mm NaCl, 10 mm NaN_3_, pH 7.4) at 298 K. [D4](trimethylsilyl)propionic acid, sodium salt (TSP, 1%) was used as internal reference. A protein concentration of 25 µm was use and the protein:ligand molar ratio was 1:50 for all the mixtures.

STD NMR experiments were acquired with shaped pulse train for saturation on f2 channel alternating between on and off resonance with 20 ms spinlock pulse applied to suppress protein signals. The acquisition was set with 65 k data points and 104 number of scans. The protein resonances were selectively irradiated by 40 Gauss pulses with a length of 50 ms, using the off‐resonance pulse frequency at 40 ppm and on‐resonance pulses at 7.5 and 0 ppm. The STD NMR spectra were carried out using a saturation time of 2 s. STD NMR effects were determined using the formula (I_0_ – I_sat_)/I_0_, where I_sat_ is the relative intensity of the STD NMR signal and I_0_ the peak intensity of an unsaturated reference spectrum (off‐resonance). The strongest STD NMR response was set to 100% while all the other STD signals were normalized to this value to provide ligands’ epitope maps.^[^ ^49^ ^]^

2D ^1^H‐^1^H NOESY experiments were acquired by using data sets of 4096 × 800 points and 100–600 ms as mixing times. Proton–proton cross‐relaxation rates were calculated by integration of cross peaks normalized against the corresponding diagonal peak. ^1^H‐^1^H distances were calculated using the following equation:

(2) $$r_{ij}=r_{ref}\;\sqrt[{6}]{\sigma_{ref}/\sigma_{ij}}$$

where *r_ij_* is the unknown distance to be estimated, *r_ref_* is the reference interproton distance, σ_*ref*_ is the cross‐relaxation rate of the NOE cross peak of interest and σ_*ij*_ is the cross‐relaxation rate of reference.^[^ ^50^ ^]^

### Fluorescence Analysis

Steady‐state fluorescence spectra were collected by means of a Fluoromax‐4 spectrophotometer (Horiba Scientific, Edison, NJ, USA). Emission spectra were recorded from 310 to 450 nm upon excitation at 295 nm. The slit widths were set to 5 nm for excitation and to 5 nm for emission wavelength. A quartz cuvette with a pathlength of 1 cm and a volume of 0.2 mL was used. The starting solution of Siglec‐7 CRD protein, prepared at a fixed concentration of 4 µm in PBS buffer (pH 7.4), was titrated by adding small aliquots of each ligand. The experiments were carried out at 10, 25, and 35 °C. For all ligands analyzed, the fluorescence intensity was quenched. The binding curve was obtained by fitting the data using nonlinear regression with a 1:1 binding model using the function described by Ribeiro et al.^[^ ^51^ ^]^ (plotting the ratio between the fluorescence intensity at each addition of ligand (*F*) and the fluorescence intensity of the protein in absence of ligand (*F*_0_) versus the total ligand concentration [L] in µm. In order to determine the enthalpy change of binding, the temperature dependence of the binding constants was evaluated. A plot of ln(*K*_b_) versus 1/*T* should give a straight line whose slope is equal to ‐Δ_b_ *H*°/*R*, allowing the determination of the enthalpy change of binding, on the assumption that Δ_b_ *H*° is independent from the temperature in the explored range.^[^ ^52^ ^]^

### Isothermal Titration Calorimetry

The ITC experiment was carried out by means of a Nano ITC‐III from TA Instruments (New Castle, DE, USA). Briefly, a 500 µm solution of ligand **1** was placed in the calorimetry vessel. Then, a solution of Siglec‐7 CRD, at the concentration of 62.5 µm was injected (injection volume of 15 µL) into the calorimetry vessel. In these conditions, the injected protein was all bound to the ligand in the calorimeter vessel. Thus, a series of similar heat peaks were obtained. The heat of dilution of the protein was evaluated by performing injection in the calorimetry vessel containing the buffer only. The temperature was set to 25 °C and the stirring speed to 250 rpm. The Δ*H_b_* ° was calculated by integration of the peaks, then subtraction of the heat of dilution, and normalizing them by the amount of injected protein.^[^ ^39^ ^]^

### MD Simulations

Glycans were generated on GLYCAM website.^[^ ^53^ ^]^ and MD simulations were performed by using AMBER 18.^[^ ^28^, ^54^ ^]^ The prmtop and inpcrd files were generated with the tLEaP module. The force fields were GLYCAM06j‐1 for carbohydrate parameters and protein.ff14SB for SLBR‐N. Complexes were properly prepared for the MD simulations by adding counter ions to neutralize the systems using the Leap module and placing them in octahedral boxes with explicit TIP3P water molecules. MD simulations were run by using the CUDA implementation of PMEMD in AMBER18.^[^ ^54^ ^]^ Minimization steps of all complexes were performed using Sander. The smooth particle mesh Ewald method was employed to describe the electrostatic attractions in the system while applying periodic boundary constraints and the grid spacing was 1 Å. The system underwent the first annealing gradually and gently over a 25‐ps period from 100 to 300 °K. Throughout 50 ps, a steady temperature of 300 °K was maintained with progressive energy minimizations. The MD coordinates were gathered to acquire 10 000 frames of the progression of MD. Using the K‐mean algorithm implemented in the ptraj module of the AMBER18 software, the trajectories were submitted to cluster analyses, in order to obtain the main representative poses. MD simulations were visualized by using VMD program.^[^ ^55^ ^]^

### RedMat

The analysis was employed considering the STD NMR effects and the best poses of the complexes between Siglec‐7 and ligands GD3 and DSGb3α3. The parameters were set as follows: NMR spectrometer frequency at 600 MHz, the concentration of protein at 25 µm, the concentration of ligand at 1000 µm, the cut‐off distance at 10 Å and the dissociation constants as derived from the fluorescence analysis. The goodness of the complexes and the accordance between the theoretical and experimental data were evaluated from the R‐factor (R‐NOE) values, 0.2 and 0.3 for ligands GD3 and DSGb3α3, respectively, and calculated using the following equation:

(3) $$R-NOE=\sqrt{\frac{\sum STD^{exp,i}-ST{D^{calc,i}}^{2}}{\sum ST{D^{exp,i}}^{2}}}\;$$

where STDexp,i is the experimental STD value for a generic proton I, while STDcalc,i is the STD value simulated with the RedMat algorithm.^[^ ^38^ ^]^

### Statistical Analysis—NMR Spectroscopy

Free induction decay (FID) and spectral editing (SED) data from 1D and 2D NMR experiments were processed using Bruker Topspin software, as detailed in the experimental procedures. For all homonuclear experiments, the data matrix underwent zero‐filling in both dimensions, resulting in a 4K × 4K point matrix. Resolution enhancement was subsequently applied in both dimensions using a cosine‐bell function prior to Fourier transformation.

### Statistical Analysis—Fluorescence Analysis

Fluorescence measurements were performed in triplicate for each data point across two independent experimental series. Data were fit using nonlinear regression analysis, as detailed in the experimental procedures.

### Statistical Analysis—Molecular Dynamics (MD) Simulations

MD simulation trajectories were analyzed by clustering to identify and analyze dominant conformational states. The clustering analysis of the MD simulation was performed using the cpptraj module, applying a k‐means clustering algorithm based on the RMSD of the selected atoms, generally the ligand, to classify different conformational states. Furthermore, to ensure computational efficiency, sieve clustering was also used, as it selects a subset of frames for clustering while maintaining statistical relevance. The algorithm was then iterated multiple times to optimize the cluster assignments, allowing us to determine the most populated conformations. This approach enables the characterization of the dominant conformations observed in the simulation, the identification of structural variability, and the extraction of key representative structures for further computational and experimental validation.

### Statistical Analysis—RedMat

The validity of 3D complex structures and the agreement between theoretical and experimental data were assessed using the R‐factor (R‐NOE), calculated according to the equation provided in the experimental section and implemented within the RedMat software.

## Conflict of Interest

The authors declare no conflict of interest.

## Supporting information



Supporting Information

## Data Availability

The data that support the findings of this study are available from the corresponding author upon reasonable request.
