# Supplementary material for: Insights into Siglec‐7 Binding to Gangliosides: NMR Protein Assignment and the Impact of Ligand Flexibility
Source: Adv Sci (Weinh). 2025 Apr 26;12(21):2415782. doi: 10.1002/advs.202415782 (PMC12140324; doi:10.1002/advs.202415782)
Supplement: Supplementary file 1 — Supporting Information [file ADVS-12-2415782-s001.pdf]

ADVANCED SCIENCE
Open Access

Supporting Information

for *Adv. Sci.*, DOI 10.1002/advs.202415782

Insights into Siglec-7 Binding to Gangliosides: NMR Protein Assignment and the Impact of Ligand Flexibility

*Cristina Di Carluccio, Luis Padilla-Cortés, Marta Tiemblo-Martìn, Giulia Roxana Gheorghita, Rosario Oliva, Linda Cerofolini, Alessandro Antonio Masi, Celeste Abreu, Hsin-Kai Tseng, Antonio Molinaro, Pompea Del Vecchio, Ondřej Vaněk, Chun-Cheng Lin, Roberta Marchetti, Marco Fragai and Alba Silipo**

Figure S1. Structure and SNFG of the gangliosides studied in interaction with Siglec-7. The aglycon moiety R is $(CH_2)_6N_3$ for ligands GD3, DSGb3α3 and DSGb3α6.

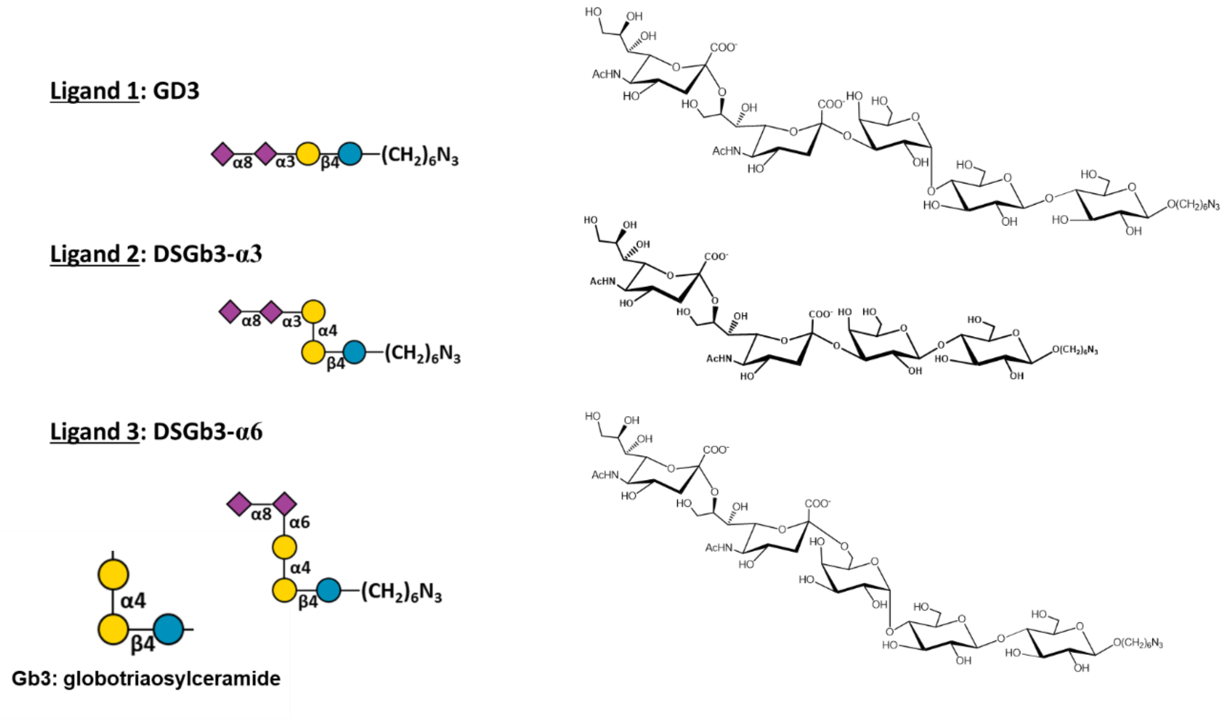

Figure S2. A) Plots of ln(K_b) vs. T-1 for the complex formation between the protein Siglec-7 CRD and ligands GD3 (black squares), DSGb3α3 (red circles) and, DSGb3α6 (blue triangles). The solid lines are the best fits to the experimental data. According to van't Hoff equation, the slopes of these lines are equal to -ΔH_b°/R, allowing the estimation of the enthalpy change of binding. B) ITC trace obtained from the titration of a solution of ligand GD3 (500 µM) with a solution of Siglec-7 CRD (62.5 µM). The experiment was performed at 25 °C in PBS buffer, pH 7.4.

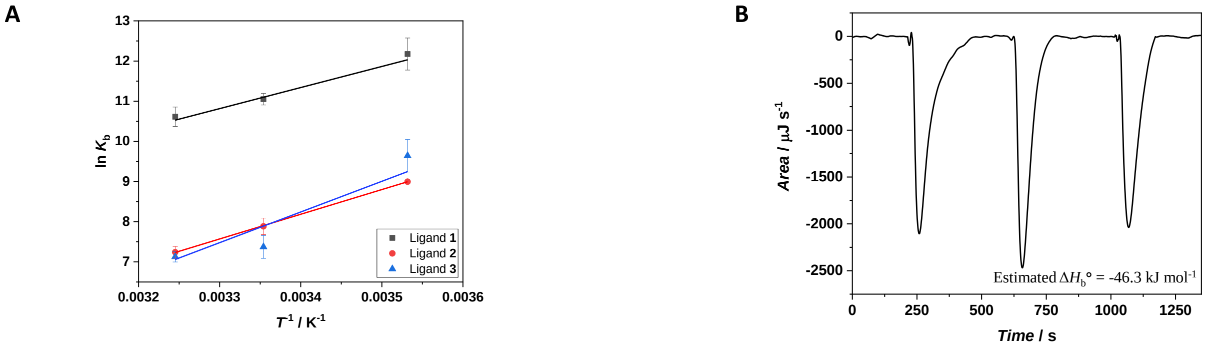

Figure S3. MD analysis of ligand GD3 in its free state and bound to Siglec-7. Maps of the dihedral angles around each glycosidic linkage of GD3 monitored along MD simulations in the free (A) and bound (B) states. The Root Mean Square Deviation (RMSD) plot was also shown to monitor the stability of the complex. C) NOESY (on the left) and tr-NOESY (on the right) NMR spectra of GD3 in the free state and bound to Siglec-7. The variation of some NOEs from the free to the bound states, including the absence of D3-K3A together with the presence of low NOE between N8 and D3 determined the bioactive conformation of GD3, showing a selection for the -g conformation around Neu5Ac-α-(2,3)-Gal glycosidic linkage.

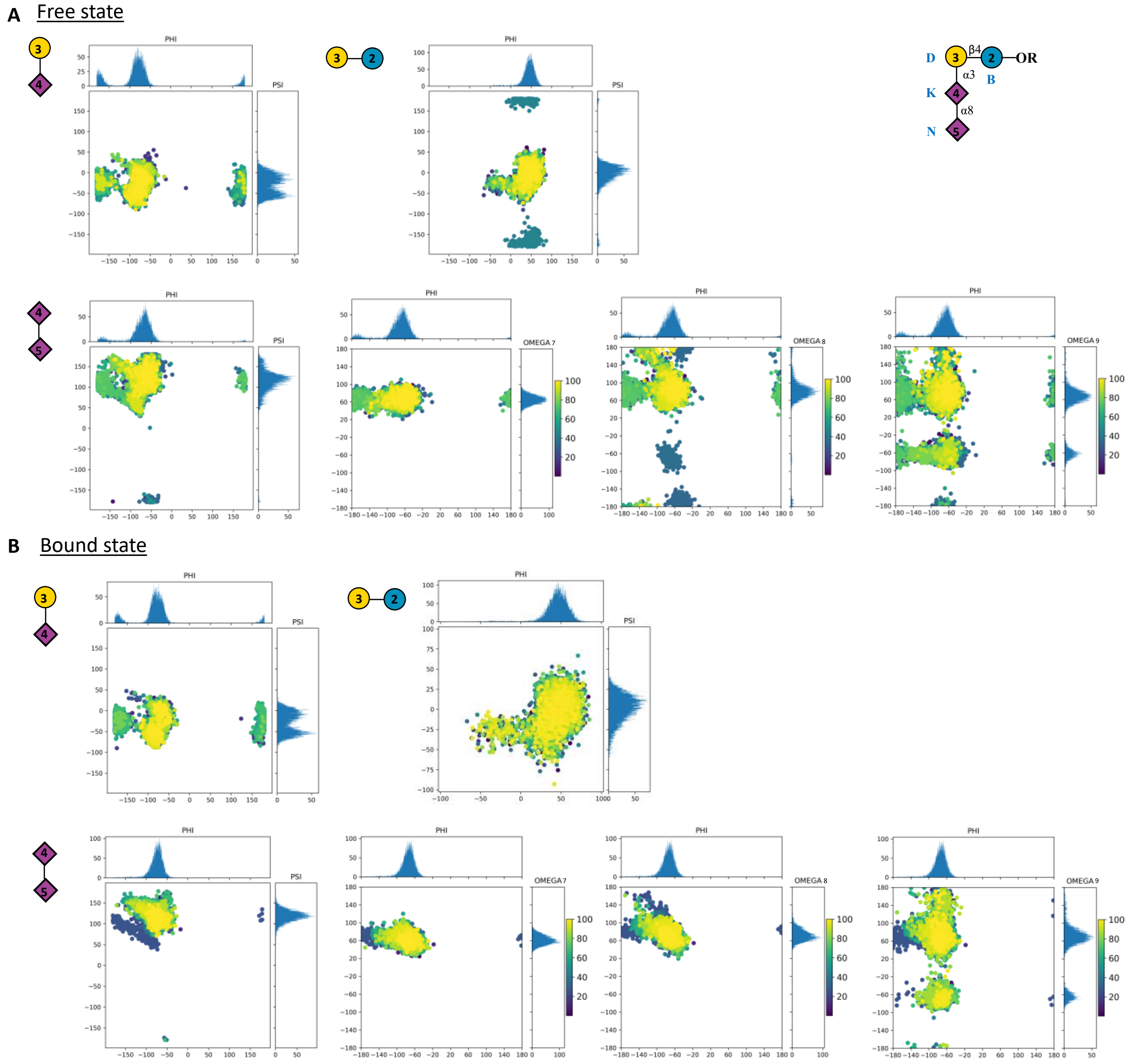

C

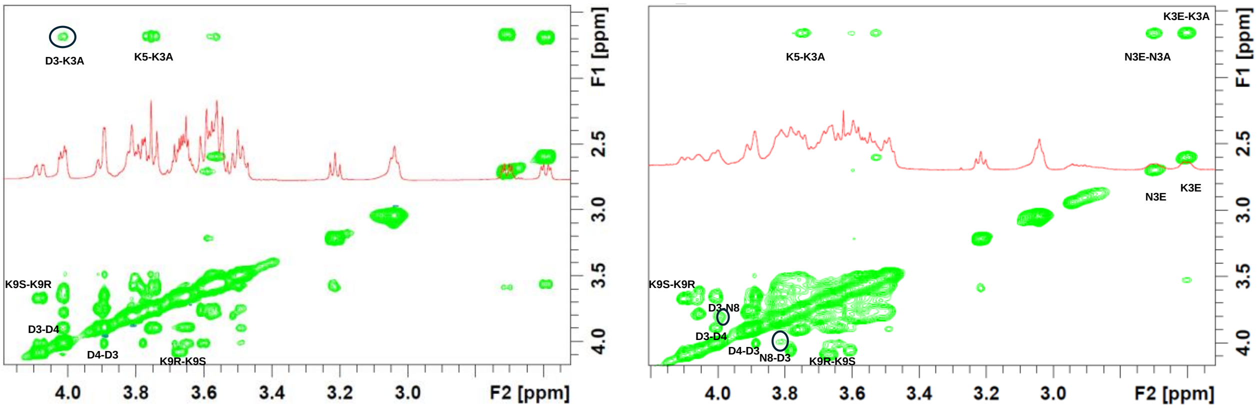
D3-K3A
K5-K3A
K9S-K9R
D3-D4
D4-D3
K9R-K9S
F1 [ppm]
F2 [ppm]
K3E-K3A
N3E-N3A
N3E
K3E
D3-N8
N8-D3

Figure S4. Maps of the dihedral angles around each glycosidic linkage of ligand **2** in the free state monitored along MD simulation.

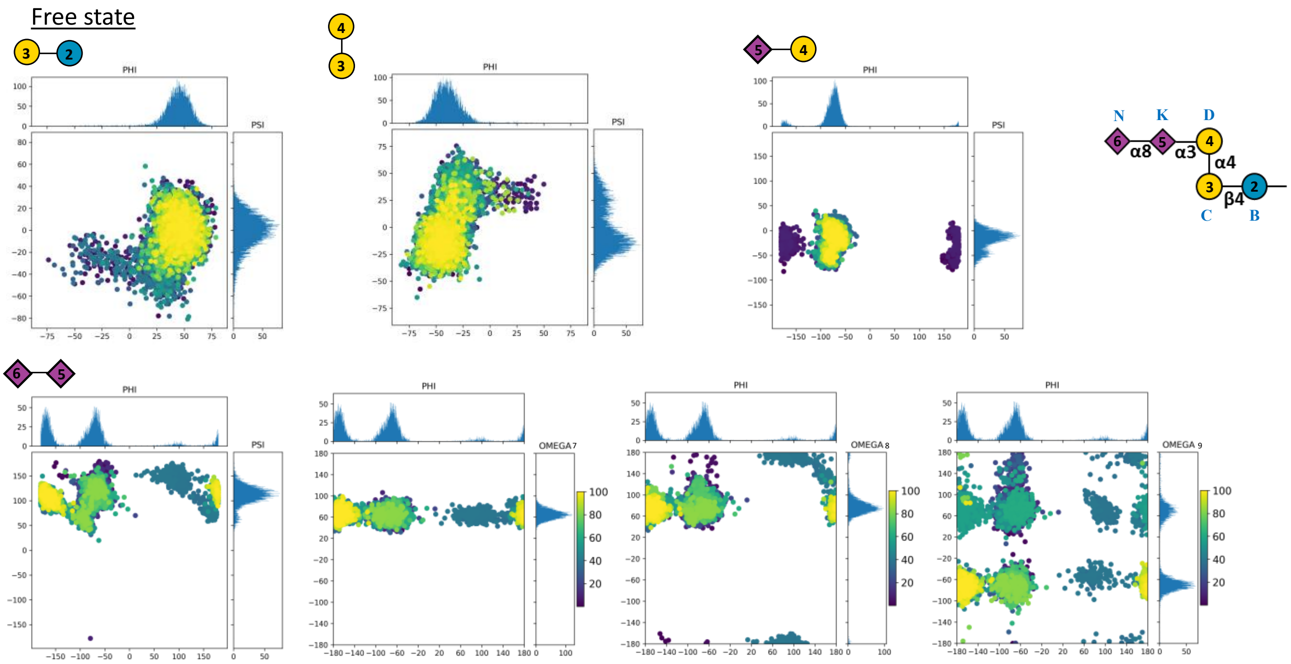

Figure S5. MD analysis of ligand DSGb3α3 bound to Siglec-7. A) Maps of the dihedral angles around each glycosidic linkage of DSGb3α3 bound to Siglec-7 monitored along MD simulation using *t* conformer as starting pose. The Root Mean Square Deviation (RMSD) plot was also shown to monitor the stability of the complex. B) Maps of the dihedral angles around each glycosidic linkage of DSGb3α3 bound to Siglec-7 monitored along MD simulation using –*g* conformer as starting pose. The Root Mean Square Deviation (RMSD) plot was also shown to monitor the stability of the complex.

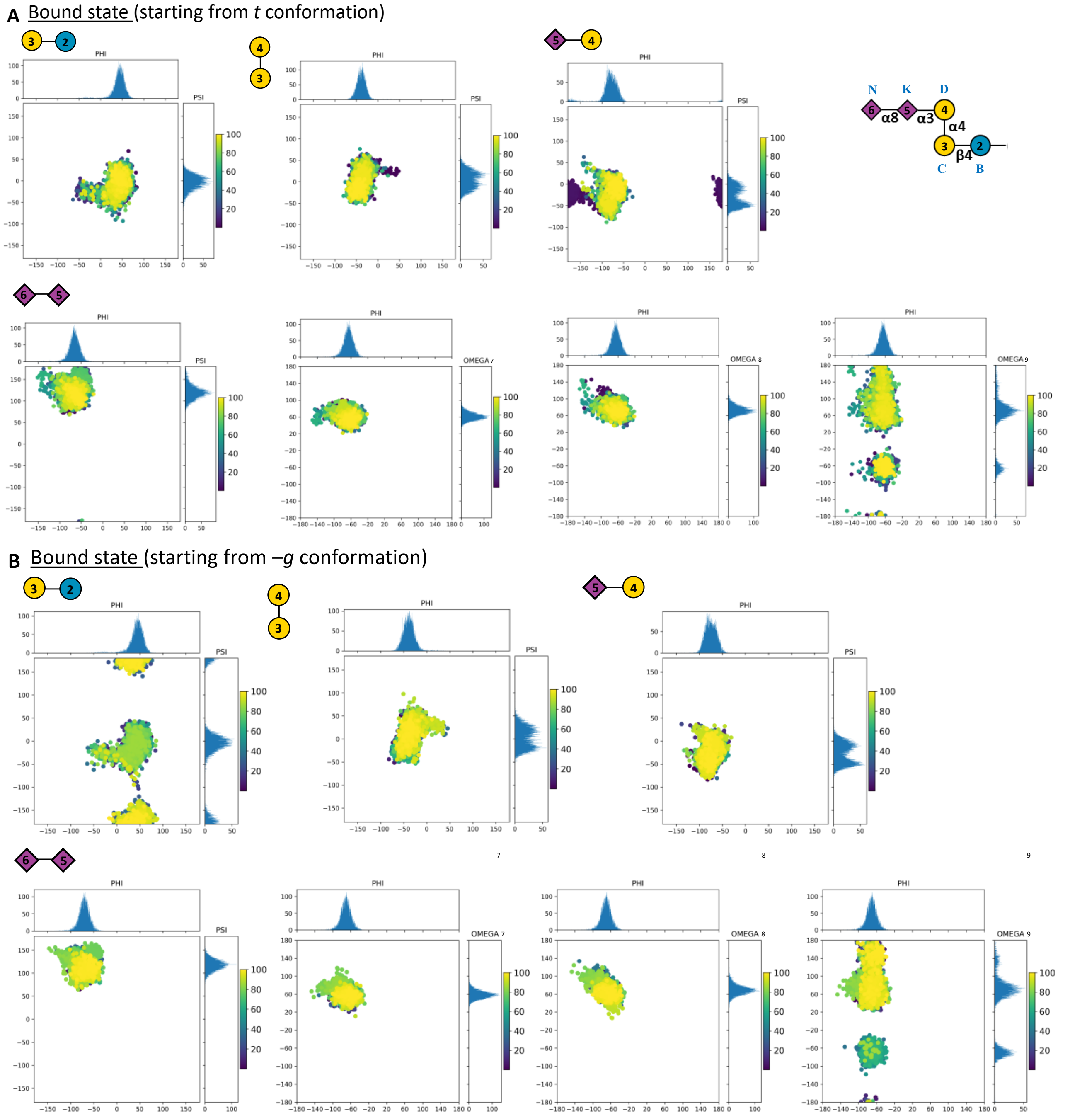

Figure S6. NOESY (left) and tr-NOESY (right) spectra of ligand DSGb3α3 in the free and bound states, respectively. The presence of low NOE contacts between K8 and N3A/N3E as well as NOE contacts between N and B residues indicate the possibility that equilibrium of the two different values of φ (-60°/-170°) around Neu5Ac-α-(2,8)-Neu5Ac occurred in solution.

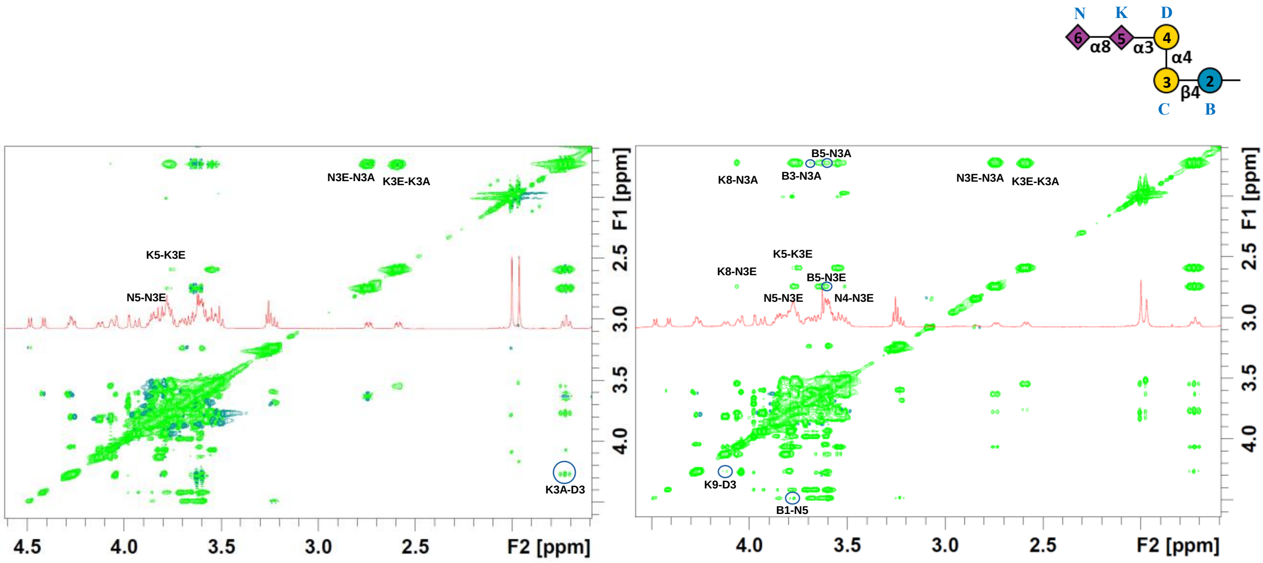

Figure S7. MD analysis of ligand DSGb3α6 in its free state and bound to Siglec-7. Maps of the dihedral angles around each glycosidic linkage of DSGb3α6 in the free state monitored along MD simulations in the free (A) and bound (B) states. C) The Root Mean Square Deviation (RMSD) plot of Siglec-7 interacting with ligand DSGb3α6 was monitored along 500 ns to determine the stability of the complex.

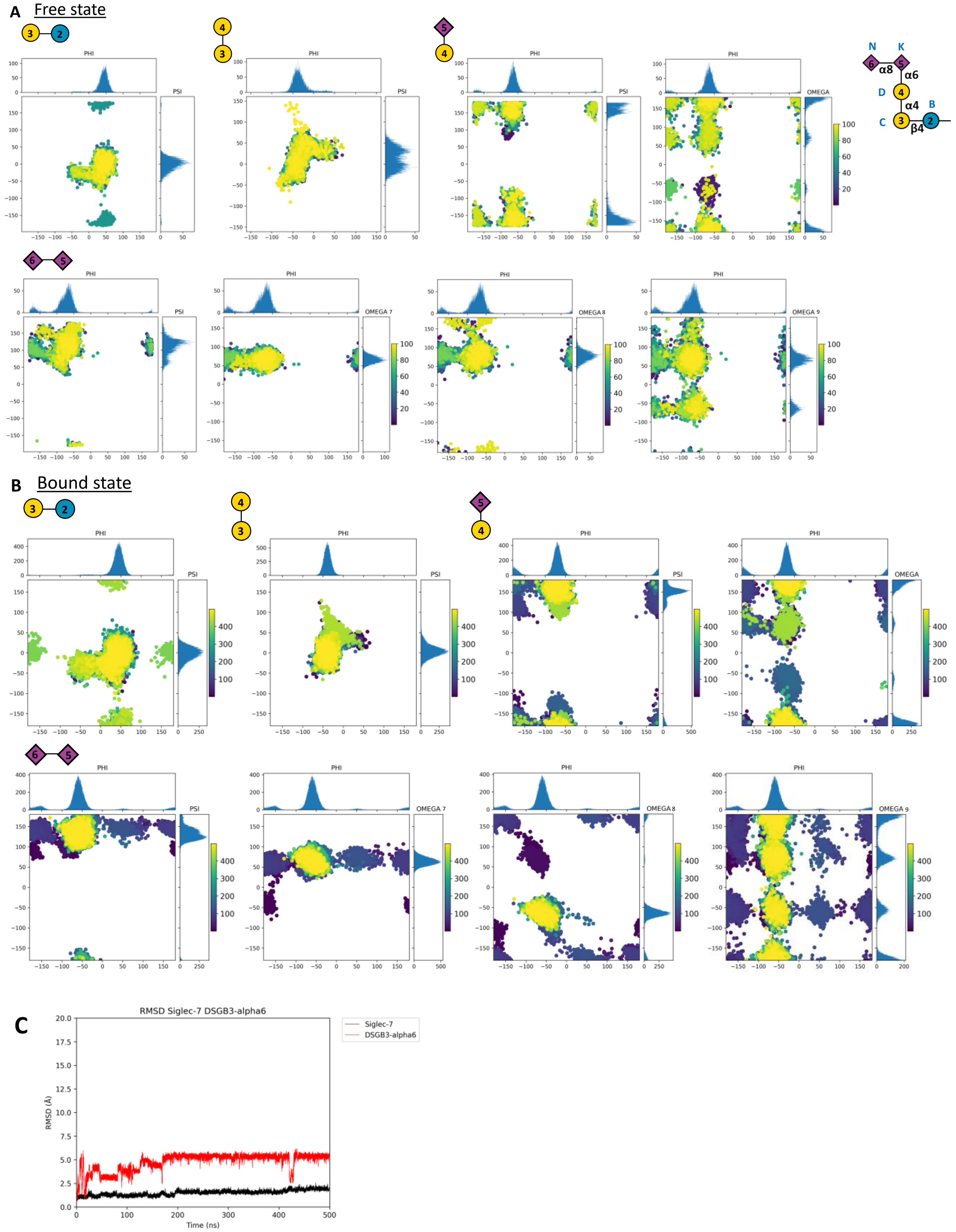

Figure S8. Conformational analysis of ligand DSGb3α6. A) NOESY and tr-NOESY spectra of ligand DSGb3α6 in the free and bound to Siglec-7, respectively. No differences were observed between the two spectra and no key NOEs were observed to determine a conformer selection. B) The difference in the multiplicity of the protons at position 6 of Gal (D residue) in the ^{13}C-HSQC determines the exclusion of the *gg* (ω=-60°) conformation around Neu5Ac-α-(2-6)-Gal glycosidic linkage.

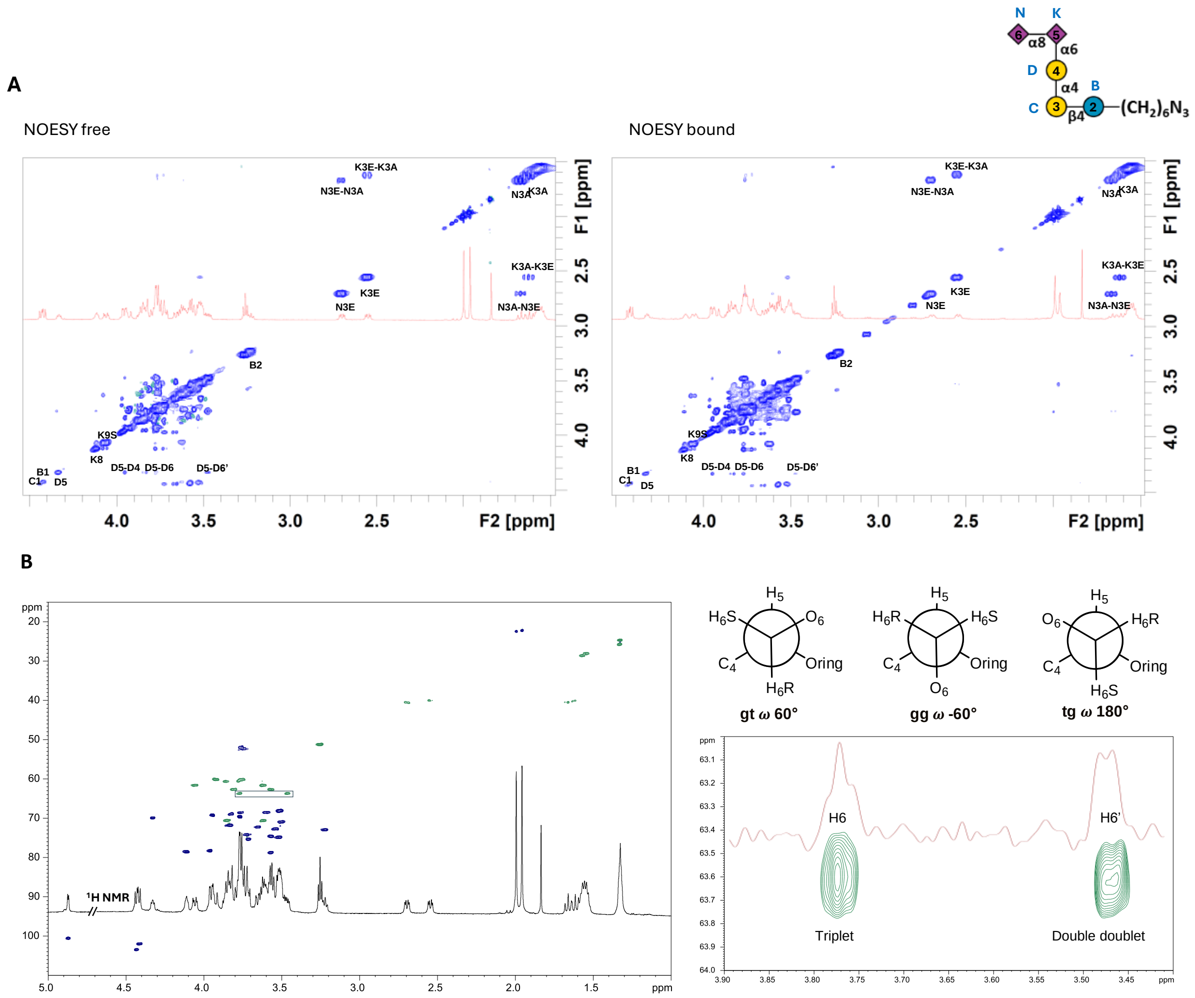

Figure S9. A comparison of the 3D complexes obtained in this study.

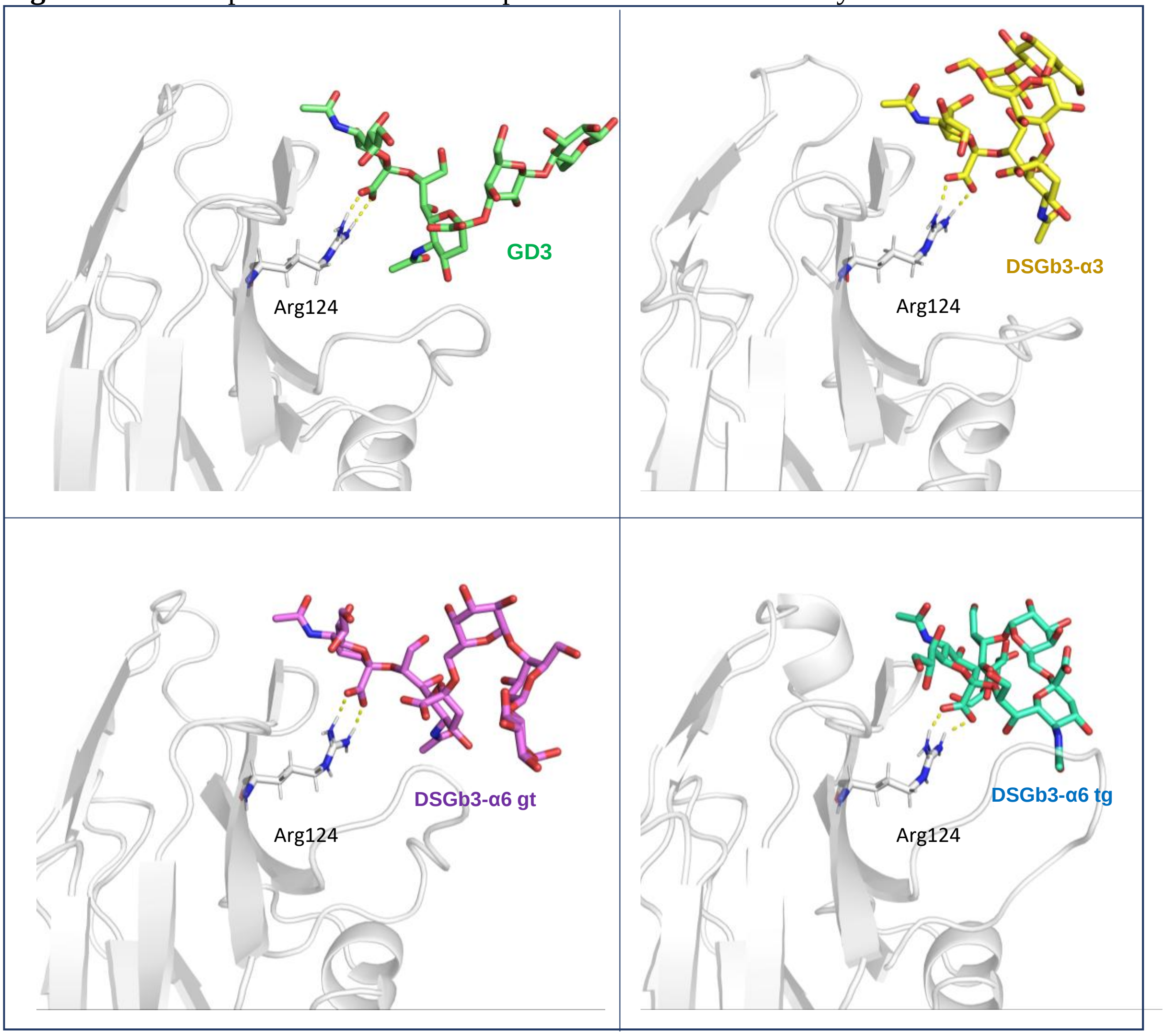

Scheme S1

A

Siglec-7 full extracellular domain (FED)

16 ITGQKSNRKD
26 YSLTMQSSVT VQEGMCVHVR CSFSYPVDSQ TDSDPVHGYW FRAGNDISWK APVATNNPAW AVQEETRDRF HLLGDPQTKN
106 CTLSIRDARM SDAGRYFFRM EKGNIKWNYK YDQLSVNVTA LTHRPNILIP GTLESGCFQN LTCSVPWACE QGTPPMISWM
186 GTSVSPLHPS TTRSSVLTLI PQPQHHGTSL TCQVTLPGAG VTTNRTIQLN VSYPPQNLTV TVFQGEGTAS TALGNSSSLS
266 VLEGQSLRLV CAVDSNPPAR LSWTWRSLTL YPSQPSNPLV LELQVHLGDE GEFTCRAQNS LGSQHVSLNL SLQQEYTGKM
346 RPVSGVLLGT HHHHHHHHG

Siglec-7 carbohydrate recognition domain (CRD)

17 MGQKSNRKD
26 YSLTMQSSVT VQEGMSVHVR CSFSYPVDSQ TDSDPVHGYW
66 FRAGNDISWK APVATNNPAW AVQEETRDRF HLLGDPQTKN
106 CTLSIRDARM SDAGRYFFRM EKGNIKWNYK YDQLSVNVTA LTHHHHHHHH

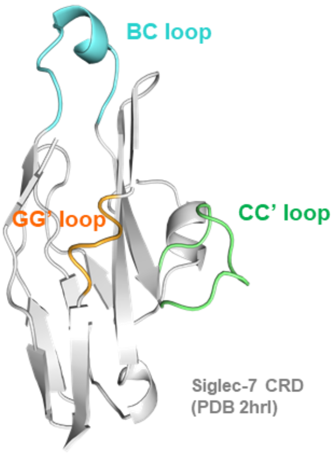

B

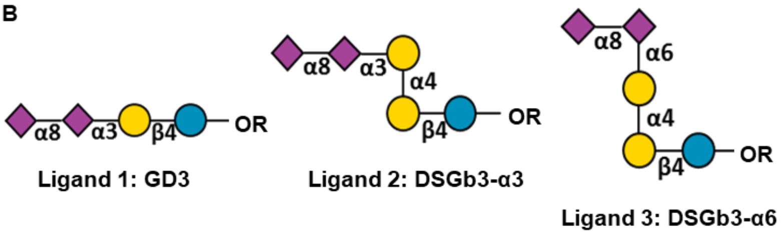

Table S1

System	$^{a}K_b$ / M^{-1}	$^{b}\Delta_bH°$ / kJ mol^{-1}	$^{c}\Delta_bS°$ / J K^{-1} mol^{-1}	$^{d}\Delta_b°G$ / kJ mol^{-1}
Siglec-7 + **1**	$(6.3±0.1)·10^4$	-43.6 ± 8.1	-54.3 ± 28.5	-27.4 ± 0.4
Siglec-7 + **2**	$(2.7±0.2)·10^3$	-51.1 ± 0.4	-106.0 ± 3.1	-19.5 ± 0.5
Siglec-7 + **3**	$(1.6±0.4)·10^3$	-63.5 ± 24.4	-151.6 ± 84.2	-18.3 ± 0.7

^{a}at the temperature of 25 °C; ^{b}obtained by means of van't Hoff analysis; ^{c}calculated using the equation $\Delta bS° = (\Delta bH° - \Delta bG°)T$; ^{d}calculated by using the equation: $\Delta bG° = -RTln(Kb)$.

Table S2

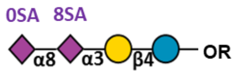

Ligand 1: GD3

Acceptor	DonorH	Donor	H-bonds
0SA@O1B	ARG_124@HH12	ARG_124@NH1	73%
0SA@O1A	ARG_124@HH22	ARG_124@NH2	70%
LYS_131@O	0SA@H5N	0SA_126@N5	49%
0SA@O8	ASN_133@H	ASN_133@N	29%
0SA@O1B	ARG_124@HH22	ARG_124@NH2	19%
0SA@O1A	ARG_124@HH12	ARG_124@NH1	18%
8SA@O4	TRP_74@HE1	TRP_74@NE1	16%

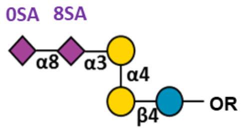

Ligand 2: DSGb3-α3

Acceptor	DonorH	Donor	H-bonds
0SA@O1A	ARG_124@HH12	ARG_124@NH1	67%
0SA@O1B	ARG_124@HH22	ARG_124@NH2	60%
0SA@O8	ASN_133@H	ASN_133@N	53%
LYS_131@O	0SA@H5N	0SA@N5	51%
0SA@O1B	ARG_124@HH12	ARG_124@NH1	32%
0SA@O1A	ARG_124@HH22	ARG_124@NH2	27%
ASN_133@O	0SA@H9O	0SA@O9	23%

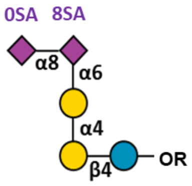

Ligand 3: DSGb3-α6

Acceptor	DonorH	Donor	H-bonds
0SA@O8	ASN_133@H	ASN_133@N	54%
0SA@O1B	ARG_124@HH12	ARG_124@NH1	51%
LYS_131@O	0SA@H5N	0SA@N5	47%
0SA@O1A	ARG_124@HH22	ARG_124@NH2	44%
0SA@O1A	ARG_124@HH12	ARG_124@NH1	42%
0SA@O1B	ARG_124@HH22	ARG_124@NH2	37%
ASN_133@O	0SA@H9O	0SA@O9	16%
